# Motives, beliefs and attitudes towards waterpipe tobacco smoking: a systematic review

**DOI:** 10.1186/1477-7517-10-12

**Published:** 2013-07-02

**Authors:** Elie A Akl, Mohammed Jawad, Wai Yim Lam, Christopher N Co, Rawad Obeid, Jihad Irani

**Affiliations:** 1 Department of Medicine, State University of New York at Buffalo ECMC-DKM C216, 462 Grider St, Buffalo, NY 14215, USA; 2 Department of Internal Medicine, American University of Beirut, Beirut, Lebanon; 3 Department of Clinical Epidemiology and Biostatistics, McMaster University, Hamilton, Canada; 4 Imperial College London, South Kensington, London, UK; 5 North Shore-Long Island Jewish Health Systems, Great Neck, NY, USA; 6 Children’s Hospital of Pittsburgh of University of Pittsburgh Medical Center, Pittsburgh, USA; 7 Faculty of Health Sciences, University of Balamand, Beirut, Lebanon

**Keywords:** Tobacco, Waterpipe, Addiction, Motives, Beliefs, Attitudes

## Abstract

**Background:**

In spite of the negative health effects of waterpipe tobacco smoking, its use is becoming more common. The objective of this study is to systematically review the medical literature for motives, beliefs and attitudes towards waterpipe tobacco smoking.

**Methods:**

We electronically searched MEDLINE, EMBASE, and the ISI the Web of Science in January 2012. We included both quantitative and qualitative studies. We selected studies and abstracted data using standard systematic review methodology. We synthesized data qualitatively.

**Results:**

We included 58 papers reporting on 56 studies. The main motives for waterpipe tobacco smoking were socializing, relaxation, pleasure and entertainment. Peer pressure, fashion, and curiosity were additional motives for university and school students while expression of cultural identity was an additional motive for people in the Middle East and for people of Middle Eastern descent in Western countries. Awareness of the potential health hazards of waterpipe smoking was common across settings. Most but not all studies found that the majority of people perceived waterpipe smoking as less harmful than cigarette smoking. Waterpipe smoking was generally socially acceptable and more acceptable than cigarette smoking in general. In Middle Eastern societies, it was particularly more acceptable for women’s use compared to cigarette use. A majority perceived waterpipe smoking as less addictive than cigarette smoking. While users were confident in their ability to quit waterpipe smoking at any time, willingness to quit varied across settings.

**Conclusions:**

Socializing, relaxation, pleasure and entertainment were the main motives for waterpipe use. While waterpipe users were aware of the health hazards of waterpipe smoking, they perceived it as less harmful, less addictive and more socially acceptable than cigarette smoking and were confident about their ability to quit.

## Background

**Figure 1:**
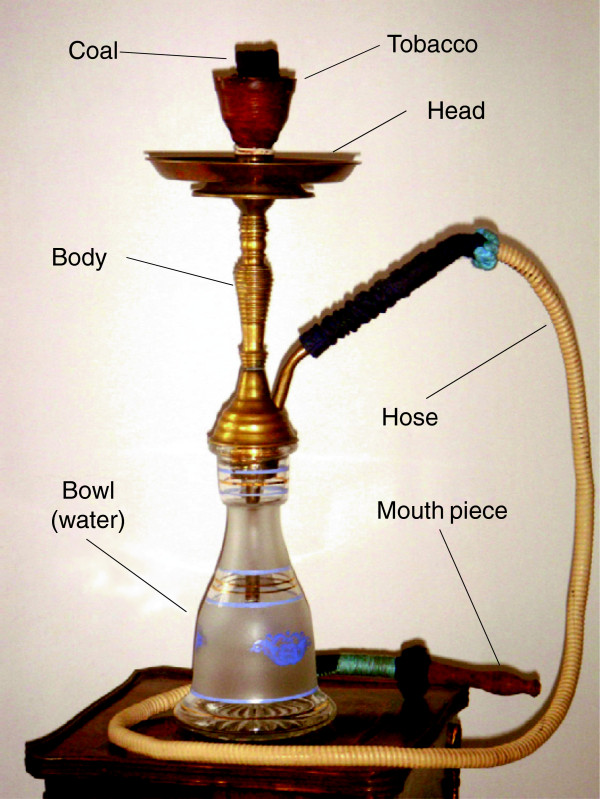
**Waterpipe device.** Annotates of the different parts of the waterpipe device.

Waterpipe tobacco smoking is traditional to region of the Middle East (Figure 1) [1]. Similarly to cigarette smoke, waterpipe smoke contains toxins that have been implicated in lung diseases (e.g. volatile aldehydes), malignant diseases (e.g. polycyclic aromatic hydrocarbons), cardiovascular diseases [e.g. carbon monoxide], and dependence (i.e. nicotine) [2].

Indeed, waterpipe tobacco smoking is associated with a number of deleterious health outcomes [3,4]. A recent systematic review has shown that it is significantly associated with lung cancer, respiratory illness, low birth weight and periodontal disease [3]. That review could not rule out an association with bladder cancer, nasopharyngeal cancer, esophageal cancer, oral dysplasia or infertility. Also cases of carbon monoxide toxicity with waterpipe smoking have been reported [5,6].

In addition, a study by Maziak et al. found that waterpipe tobacco smoking is likely to be associated with the risk of dependence [7]. It is also possible that it can serve as a gateway to initiate cigarette initiation or as a replacement for cigarette smoking among quitters [8].

In spite of the negative health effects of waterpipe tobacco smoking, its use is becoming more common. A recent systematic review found that the prevalence of waterpipe use is alarmingly high among school students and university students in Middle Eastern countries and among groups of Middle Eastern descent in Western countries [9].

Misconceptions about the harm caused by waterpipe smoking might be contributing to the increased prevalence of waterpipe tobacco smoking. While a number of studies have addressed motives, beliefs and attitudes, we identified no systematic review attempting to summarize their results.

The objective of this study was to systematically review the medical literature for motives, beliefs and attitudes waterpipe tobacco smoking.

## Methods

### Eligibility criteria

We included both quantitative and qualitative studies that assessed the motives, beliefs or attitudes about waterpipe use. We excluded studies that did not distinguish waterpipe smoking from other forms of smoking. We excluded studies or data about forms of tobacco smoking other than waterpipe even if conducted among waterpipe users. We also excluded studies reported as abstracts and for which we could not identify a full text.

### Search strategy

In January 2012, we searched the following electronic databases: MEDLINE (1950 onwards; access via OVID), EMBASE (1980 onwards; access via OVID), and ISI the Web of Science. We did not use language restrictions. The search strategy was in part based on that of a systematic review on interventions for waterpipe smoking cessation, on a review of eligible papers, an Internet search for the synonyms of waterpipe and input from two medical librarians (Additional file 1) [10]. In addition, we reviewed the reference lists of included and relevant papers and used PubMed’s 'Related Articles' function.

### Selection process

Two reviewers independently screened the title and abstract of identified citations using a standardized screening guide. We obtained the full texts of citations considered as potentially eligible by at least one of the two reviewers. Then, the two reviewers screened the full texts for eligibility in a duplicate and independent manner using a standardized and pilot tested screening form. They resolved their disagreements regarding final eligibility by discussion or with the help of a third reviewer.

### Data abstraction

Two reviewers independently abstracted data from each eligible report using a standardized and pilot tested data abstraction form. They resolved disagreements with the help of a third reviewer. Abstracted data related to:

1. Methodology: sample frame, sampling method, recruitment method, and administration method;

2. Methodological quality: sample size calculation, sampling type, validity of survey tool, pilot testing, and response rate;

3. Population: country, participant characteristics, setting, numbers for subjects in the study.

4. Motives, beliefs and attitudes towards waterpipe tobacco smoking. We considered as beliefs what certain reports labeled as knowledge (e.g., knowledge that waterpipe contains addictive substances).

### Data analysis

We calculated the kappa statistic to evaluate the agreement between the two reviewers assessing full texts for eligibility. Two authors (EAA, CC) reviewed in an iterative process the results of individual studies to identify common themes and develop a structure for reporting the results. We synthesized data qualitatively and stratified the results by Western vs. Middle Eastern societies and where applicable to whether the setting was a school, university or a community.

## Results

### Description of included studies

**Figure 2:**
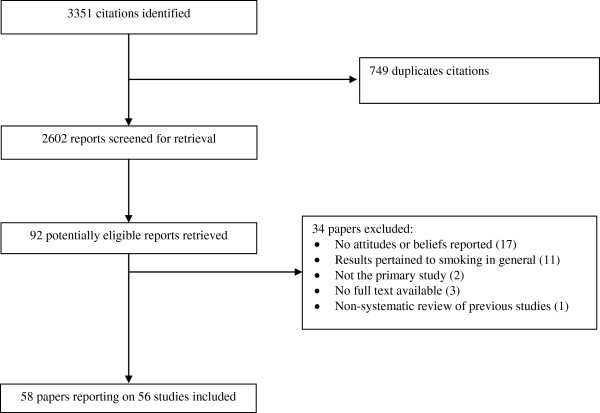
**Study flow diagram.** Shows the flow of the studies in the process of screening and selection.

Figure 2 shows the study flow. Of 92 potentially eligible papers, we excluded 34 for the following reasons: No attitudes or beliefs reported (17), results pertained to smoking in general (11), not the primary study (2), no full text available (3), and non-systematic review of previous studies (1). We included 58 papers reporting on 56 studies. Additional file 2 details the characteristics of each study.

Of the 56 included studies, 54% were conducted in Middle-Eastern countries while the rest were conducted in Western countries. The distribution of the setting of these studies was as follows: a school (18%), a university (36%), and a community (44%). Two studies included respondents from both university and community settings [11,12]. One study recruited only pregnant women [13]. Three studies were qualitative [14-16], while four studies included a qualitative component [17-20].

### Methodological quality of included studies

Additional file 2 details the methodological quality of each study. Eleven studies (20%) reported sample size calculation. Of 46 studies (82%) reporting the type of sampling, 52% used random sampling. Nineteen studies (34%) used a valid survey tool. Seventeen studies (30%) reported pilot testing their tool. Thirty eight studies (38%) reported their response rates, which varied from 18% to 100%.

### Synthesis of results

Additional file 2 details the results of each study individually. We qualitatively synthesized these results according to the following themes: motives, perceived health hazards, perceived harms relative to cigarette smoking, perceived social acceptability, perceived addictive properties, attitude regarding quitting, and perceived ability to quit. None of the studies was consisted of a national survey.

### Motives

#### Western societies

Ten studies assessed the motives for waterpipe smoking in Western societies [12,16,19,21-27].

Four studies were conducted in community settings in the USA. The main identified motives were socializing with family and friends, taste [21,25], and relaxation [21,27]. Additional motives included enjoyment of the smell [21], influence of friends and family, fashion, and loneliness [19].

Four studies recruited university students in the USA. The commonly reported motives were social gathering [22,23], flavor and smell [22,26], relaxation [12,23], peer influence [12,23,26], and experimentation/curiosity [12,26]. In one study, motives included liking the way the waterpipe is crafted, and the convenience of waterpipe cafes nearby [26].

In a qualitative study among Canadian and English students, respondents perceived waterpipe smoking as ‘exotic’ and ‘intimate’. Most respondents were of Arabic background and considered waterpipe smoking as a means to express their Arab heritage. Non-Arabic respondents found waterpipe smoking to be an affordable relaxing novelty; the tobacco’s fruit flavors with the inhalation and exhalation of large quantities of smooth smoke provided a ‘relaxing appeal’ and made it more attractive than cigarettes [16].

One study recruited high school students from Johannesburg, South Africa [24]. Motives included the absence of alternative recreation (46%), relaxation (28%), peer pressure (14%), and addiction (7%) [24].

#### Middle Eastern societies

Nineteen studies reported the motives in Middle Eastern societies [14,15,28-43].

Six studies were conducted in community settings, mainly among café patrons.

•In Egypt, café patrons preferred waterpipe over cigarettes because of habit (27%), less smoking hours (24%), and less harm (21%) [31].

•Iraqi male café patrons used waterpipe mainly for entertainment (70%), and less frequently because of addiction (13%) or as a way to quit cigarettes (9%) [29].

•In Lebanon, heavy waterpipe smokers reported the following reasons for using waterpipe: increased availability of cafes, increased affordability, innovations in designs of waterpipe apparatus and tobacco flavors, sensory qualities (taste, smell, sight of smoke, sounds of bubbling), and positive media portrayal of waterpipe [15].

•In Syria, smokers considered waterpipe as a pleasurable and entertaining social experience fostering a sense of togetherness as well as cultural identity [14,28]. This was in contrast to cigarette smoking, which was often perceived as a ‘mundane, oppressive, personal addiction’ that dominated their lives in exchange for temporary relief from anxiety [14,28].

•In Turkey, the most commonly reported reason for waterpipe use was peer influence (35%); less common reasons included curiosity (18%), influence of family members (12%), relaxation (9%), taste (7%), influence of social environment (4%), imitation (4%), and as replacement for cigarettes (2%) [30].

Nine studies assessed the motives of university students.

•In Egypt, female medical and undergraduate university students preferred waterpipe over cigarette smoking because it was fashionable (20%), “less harmful” (19%) and allowed them to be with their friends (18%) [35].

•Four studies were conducted among Iranian university students [34,37,39,40]. Commonly cited motives included: relieving stress, anxiety, anger and depression [39,40]; fun and socializing [34,37]; and pleasure [39,40]. In one study, participants thought waterpipe smoking improves concentration, self-efficacy, social acceptability, and to become mature, and popular [39]. Additional motives were peer pressure [40], and curiosity [34].

•Among Kuwaiti students, nonsmokers and cigarette smokers believed it was social pressures that encouraged people to smoke waterpipe, whereas waterpipe smokers did not believe that was true [36].

•In Pakistan, university students initiated waterpipe smoking because of curiosity (61%), pleasure-seeking (47%), and peer pressure (23%) [32].

•In Syria, the most common positive perceptions of waterpipe by university students were its smell and taste [33].

•Turkish medical and engineering students reported using waterpipe mainly because of enjoyment (72%) but also because of peer pressure (12%) [38].

Four studies evaluated motives among adolescents:

•In Iran, and among youngsters aged 12 to 20, 92% of male smokers and 97% of female smokers reported using waterpipe as a means of entertainment, hospitability, and as a symbol of fashion [42].

•In Lebanon, adolescents from urban neighborhoods stated that those who had friends who smoke and whose friends encouraged them to smoke were more likely to continue smoking than those who did not [41]. In a second study, motives for waterpipe smoking included: expression of manhood, families encouraging their children to use waterpipe at home in social gatherings, and as a way to “forget problems” [20].

•In Israel, school students reported smoking waterpipe for the pleasure and for the intimacy that it added to their informal gatherings [43].

### Perceived health hazards

#### Western societies

Seven studies all conducted in the USA found consistent results [18,22,23,25,26,44,45]. A large majority of respondents were aware of the negative health effects of waterpipe smoking [18,22,23,25,26,44,45]. In one study, 92% of participants believed it can cause respiratory problems, 69% believed it has cardiovascular effects, and 69% felt it can cause cancer [23]. One study found that students who received information about harms and exposures of waterpipe smoking reported greater perceived personal health risk and expressed more worry compared to those who did not [26].

#### Middle Eastern societies

Ten studies conducted in Middle Eastern countries found that people were aware of the potential health hazards of waterpipe smoking [13,20,30,34-36,46-49].

•In Egypt, 84% of male university students believed waterpipe smoking to be hazardous [49].

•Of the Jordanian university students interviewed regarding harmful effects of waterpipe smoking, 37% believed it causes respiratory disease, 35% cancer, 20% cardiovascular disease, and 6% mouth disease [48].

•In Lebanon, male adolescents were aware of the negative health effects of waterpipe smoking but nevertheless continued to smoke [20]. Pregnant women interviewed in Lebanon believed that waterpipe contains addictive substances (45%), produces harmful gases (39%), contains carcinogens (42%), affects the fetus (74%) and the newborn (71%) [13].

•In Iran, among students from a health sciences university, 73% of current and 68% of occasional waterpipe users were aware of the health hazards of waterpipe smoking [34].

•In Kuwait, non-smokers held significantly stronger beliefs about the negative health effects of waterpipe smoking, compared to waterpipe and cigarette smokers who were least likely to believe in them [36].

•In Aleppo, Syria, university students and waterpipe café customers identified respiratory effects, cancer and cardiovascular disease as the top health hazards of waterpipe smoking [47].

•In Karachi, Pakistan, 56% of university students believed that waterpipe contains significant amounts of tobacco, 53% believed that it can cause cancer, and 73% believed it can cause respiratory problems [35]. A second Pakistani study assessed the impact of interactive health sessions regarding waterpipe smoking among adolescents. Belief in health hazards was higher for the pre-test group compared to the post-test group for cardiovascular effects (24% vs. 10%) and cancer risk (41% vs. 37%) but not for respiratory effects (70% vs. 72%), oral infections (12% vs. 17%), and other bodily effects (18% vs. 23%) [46].

•In Turkey 23% of waterpipe smokers in cafes, believed that waterpipe smoking could spread communicable diseases [30].

### Perceived harms relative to cigarette smoking

#### Western societies

We identified 15 studies conducted in the USA, 9 in school or university settings [22,27,44,50-55], and six in community settings [12,18,21,45,56,57].

In 5 of the 9 studies conducted in American school or university settings, the majority of respondents (52 to 77%) felt that waterpipe smoking was equally or more harmful than cigarette smoking [22,52-55]. In one study, these percentages were higher among non-smokers compared to smokers [52]. In four studies, the majority of respondents felt waterpipe smoking was less harmful than cigarette smoking [27,44,50,51]. One of these studies found that ever-users of tobacco were more likely to believe that waterpipe smoking is less risky than cigarettes [27]. Students believed that waterpipe smoking was less harmful because there is little or no nicotine, there are fewer chemicals, and water filters the smoke [50].

In the 6 studies conducted in American community settings, the majority of respondents perceived waterpipe smoking as less harmful than cigarette smoking [12,18,21,45,56,57]. They believed that waterpipe contained less nicotine, that the water has filtering properties, and that switching from cigarettes to waterpipe would reduce their health risks [18,21,56]. In one study, the flavor and the smell were cited as indicators that waterpipe is safer than other tobacco products [18].

In a study of British university students smokers of waterpipe, 68% of those who thought waterpipe is bad for health believed it was less damaging than cigarettes [58]. In a qualitative study conducted in Canadian and English waterpipe cafes, respondents thought that waterpipe was less harmful than cigarettes [16]. They also reported that the lack of media campaigns about waterpipe smoking implied that they must be safer [16]. One study from Australia (reported in 2 papers) found that 81% of respondents to telephone surveys among Arab-speaking residents perceived waterpipe smoking as more harmful [59,60]. A study from Johannesburg South Africa found that 53% of high school students using waterpipe think it is less harmful than cigarettes [24].

#### Middle Eastern societies

Out of 18 studies, 12 found that the majority of respondents believed that waterpipe smoking was less harmful than cigarette smoking [13,14,29,30,32,34,35,43],[46-48,61]. Reasons behind this perception included: filtering effect of water [29,32], less nicotine content than cigarettes [32], detoxification of the produced smoke by fruit flavors [32], and production of less harmful gases and less carcinogens [13]. In 6 studies the majority of respondents believed that waterpipe was as or more harmful than cigarettes [33,38,41,49,62,63].

### Social acceptability

#### Western societies

60% to 95% of respondents to 4 survey studies considered waterpipe smoking very socially acceptable [44,50,53,58]. In one survey study, respondents stated that waterpipe smoking is the most socially acceptable form of tobacco [21]. In another survey, 78% of respondents thought it will become more popular in the next five years [21]. The social attribute to waterpipe was considered important both in its initiation and continuous use [12,16,64]. In one study the majority (61%) of parents who knew about their children’s use of waterpipe disapproved it [22].

#### Middle Eastern societies

The social acceptability of waterpipe smoking appears to vary by country. In Syria and Pakistan, family attitudes towards waterpipe smoking were mostly either neutral or positive, particularly compared to cigarette smoking [14,32,33,46,65]. Three studies from Syria reported that family members are generally more tolerant or permissive of female relatives smoking waterpipe than female relatives smoking cigarettes [33,47,66]. In contrast, respondents from Lebanon and Egypt mostly felt that family members disapproved of waterpipe smoking [20,31,61]. In another study from Syria, a majority of participants thought that smoking waterpipe is religiously unacceptable [63]. The social attribute of waterpipe was considered important its initiation in Kuwait [36]. In one study from Lebanon, participants reported that waterpipe smoking was spreading fast due to its social acceptance compared to cigarette smoking [20].

### Perceived addictive properties

#### Western societies

52 to 79% of participants in three studies felt that they were less addicted to waterpipe than to cigarette smoking [21,53,56]. Two of these studies reported that almost 90% of respondents did not consider themselves ‘hooked’ or dependent on waterpipe smoking [21,56]. In a survey of freshmen university students in the USA, the majority of respondents believed there was a low or no chance to becoming addicted when using waterpipe on their own (54%) or socially (67%) [44]. In a survey of university students who smoked waterpipe, respondents believed that addictive effect of waterpipe use is unlikely because of its occasional occurrence [12].

#### Middle Eastern societies

In one study the majority of respondents believed waterpipe smoking is not addictive [30]. The majority of respondents to 3 studies felt that waterpipe smoking was less addictive than cigarette smoking [11,32,62]. However, one qualitative study reported that frequent waterpipe smokers felt addicted in a similar way to cigarette smokers [14].

### Perceived ability to quit

In both Western and Middle-Eastern societies, most respondents (79 to 98%) had a high degree of confidence that they could quit waterpipe smoking at any time [21,23,47,51,56]. Of these, one study reported that 80% of smokers felt that quitting waterpipe was easier than quitting cigarette smoking, and that 62% of those interested in quitting waterpipe did not expect any challenges in doing so [65]. However, in one study in Syria, 57% of waterpipe smokers believed that quitting smoking is difficult [63].

### Attitude regarding quitting

Five studies conducted in the USA reported percentages of waterpipe smokers interested in quitting that varied from 26% to 53% [21,22,25,50,51].

Studies conducted in Middle Eastern countries reported percentages of waterpipe smokers interested in quitting as follows: 50% in Iraq [29], 62% in Egypt [31],, 21% in Turkey [38], 49% in Syria [56], and 64% in Lebanon [61].

Three studies conducted in Syria reported that participants were more interested in quitting cigarette than waterpipe smoking [14,28,65]. Also in Syria, university students showed more interest in quitting this habit compared to waterpipe café customers (41% vs. 28%) [47]. In two studies in Pakistan and Kuwait, respectively 28% and 51% of respondents who were interested in quitting had previously attempted to quit [36,46]. In Egypt, the majority of students had a desire to quit after one and five years (72% and 44%), and 77% felt that smokers should be informed about the possibility to quit [49].

The main reason cited for quitting waterpipe smoking was health concern [50,65]. Other reasons included no longer liking waterpipe smoking, smoking cigarettes instead, and using other tobacco products [50]. As for the challenges to quitting waterpipe smoking, respondents cited boredom, socializing with friends who were smoking, and addictiveness [47,65].

## Discussion

In summary, the main motives for waterpipe use were socializing, relaxation, pleasure and entertainment. Additional motives reported by university and school students were peer pressure, fashion, and curiosity. Expression of cultural identity was a specific motive for people in the Middle East and for people of Middle Eastern descent in Western countries. Waterpipe smoking was generally socially acceptable. In Middle Eastern societies, it was particularly more acceptable for women’s use compared to cigarette smoking. Waterpipe users were aware of the health hazards of waterpipe smoking, but perceived it as less harmful, and less addictive than cigarette smoking. While users were confident about their ability to quit, the willingness to quit varied across settings.

This study has a number of strengths. To our knowledge, this is the first systematic review of motives, beliefs and attitudes toward waterpipe tobacco smoking. In addition, we followed the Cochrane Collaboration rigorous methodology in performing this review. Also, our findings cover both Middle Eastern to Western countries, and different settings (i.e., school, university and community). Finally, analyzing the results according to the geographical areas (Western vs. Middle Eastern) allowed us to identifying culture specific motives, beliefs and attitudes. The major limitation of this review relates to the methodological shortcomings of the included studies (e.g., the use of non-standardized tools to measure motives, beliefs and attitudes). However, the overall consistency of findings increases our confidence in the results.

The findings of this review help explain the profile of the waterpipe epidemic: school students and university students, Middle Eastern countries, and groups of Middle Eastern descent in Western countries [67]. Indeed peer pressure, curiosity, and the “fashion” appear to be specifically influential with students. The high prevalence in Middle Eastern countries is apparently related to the social acceptance, particularly for women’s use [68], and the belief that the practice is not as harmful as cigarette smoking. An additional factor affecting groups of Middle Eastern descent in Western countries is the “cultural heritage” assigned to waterpipe.

## Conclusion

### Implications for public health policy

Public health authorities should scale-up efforts to combat the waterpipe epidemic. Indeed, respondents to one survey reported that the lack of media campaigns about waterpipe implied that they must be safer [16]. Public health interventions should aim to “deglamourize and renormalize” waterpipe smoking [10] which is perceived as a “fashion”, and as an expression of cultural identity. In addition, interventions should take into account the role of social marketing (as opposed to industry marketing such as advertisements) [9]. Also, public health interventions need to be tailored to the target group. For example, campaigns to improve awareness of the harms of waterpipe smoking might be effective in Middle Eastern societies where waterpipe smoking smokers believe it to be less harmful than cigarette smoking.

### Implications for research

There is a need to develop and validate a survey instrument for measuring motives, beliefs and attitudes related to waterpipe smoking [4]. Such a tool would be useful to identify factors that could modify the effects of future interventions designed for waterpipe smoking cessation. It would also be useful to follow longitudinally the trends in motives, beliefs and attitudes in order to assess and guide public health interventions.

## Competing interests

The authors declare that they have no competing interests.

## Authors’ contributions

EAA contributed to drafting the protocol, designing the search strategy, developing the forms, screening, data abstraction, data analysis, and drafting of the manuscript. MJ and WYL contributed to data abstraction, data analysis, and drafting of the manuscript. CNC an RO contributed to data abstraction. JI contributed to drafting the protocol and designing the search strategy. All authors revised the article critically for important intellectual content and approved its final version.

## Supplementary Material

Additional file 1 Electronic search strategies. Click here for file

Additional file 2 Included studies on motives, beliefs and attitudes regarding waterpipe smoking, organized by country of conduct (western and non-western). Click here for file
