# Supplementary material for: Motives, beliefs and attitudes towards waterpipe tobacco smoking: a systematic review
Source: Harm Reduct J. 2013 Jul 2;10:12. doi: 10.1186/1477-7517-10-12 (PMC3706388; doi:10.1186/1477-7517-10-12)
Supplement: Additional file 1 — Electronic search strategies. [file 1477-7517-10-12-S1.doc]

**MEDLINE** (1950 onward)

Waterpipe*.mp.

water pipe*.mp.

shisha*.mp.

sheesha*.mp.

hooka*.mp.

huqqa*.mp.

guza*.mp.

goza*.mp.

narghil*.mp.

nargil*.mp.

arghil*.mp

argil*.mp

(hubbl* adj3 bubbl*).mp.

ghalyun.mp

okka.mp

kalian.mp

Lula.mp

Lulava.mp

or/1-18

**EMBASE** (1980 onward)

Waterpipe*.mp.

water pipe*.mp.

shisha*.mp.

sheesha*.mp.

hooka*.mp.

huqqa*.mp.

guza*.mp.

goza*.mp.

narghil*.mp.

nargil*.mp.

arghil*.mp

argil*.mp

(hubbl* adj3 bubbl*).mp.

ghalyun.mp

okka.mp

kalian.mp

Lula.mp

Lulava.mp

or/1-18

**ISI the Web of Science**

(waterpipe* OR "water pipe*" OR shisha* OR sheesha* OR hooka* OR huqqa* OR guza* OR goza* OR narghil* OR nargil* OR argil* OR arghil* OR (hubbl* SAME bubbl*) OR ghalyun* OR okka* OR kalian* OR Lula* OR Lulava*) AND (smoking OR smoke OR health OR disease OR cancer* OR malignan* OR lung* OR pulmonary OR heart OR cardiac OR vascular OR stroke) (in Title or Topic)
