# Supplementary material for: Motives, beliefs and attitudes towards waterpipe tobacco smoking: a systematic review
Source: Harm Reduct J. 2013 Jul 2;10:12. doi: 10.1186/1477-7517-10-12 (PMC3706388; doi:10.1186/1477-7517-10-12)
Supplement: Additional file 2 — Included studies on motives, beliefs and attitudes regarding waterpipe smoking, organized by country of conduct (western and non-western). [file 1477-7517-10-12-S2.docx]

**Additional file 2:** Included studies on motives, beliefs and attitudes regarding waterpipe smoking, organized by country of conduct (western and non-western)

| **Study** | **Methodology** | **Methodological qualities** | **Population and Setting** | **Results** |
| --- | --- | --- | --- | --- |
| **Western countries** | | | | |
| **Abughosh**  **2011 [**[**1**](#_ENREF_1)**]** | - Sampling frame: students of the University of Houston (N: not reported) - Sampling method: not reported - Recruitment method: email - Administration method: internet, self-administered survey | - Sample size calculation: no - Sampling type: not reported - Validity of tool: previously reported validated tool - Pilot testing done: not reported - Response rate: not reported | - Country: USA - Participants: students of the University of Houston (UH) (62.01% over 22 years old, 50.3% male, 45% white non-Middle Eastern, 18.2% Latino/ Hispanic, 8.4% Middle Eastern, 8.2% Indian/ Pakistani) - Cigarette smokers:   Ever- 82.19%   - Cigar smokers:   Ever- 76.84%   - Waterpipe smokers:   Ever- 51.77%  Current monthly- 19.11%  Current weekly- 4.57%   - Setting: university (internet), Feb to Mar 2011 - N sampled- not reported - N participated- 2,204 - N analyzed- 1,141 | - 75.8% believed hookah was harmful to health (24.2% believed it wasn’t) - 57.5% believed it was less harmful than cigarettes (42.5% believed it was as or more harmful) - 67.1% thought there was a low or no chance to get addicted when using hookah socially (32.9% felt there was a medium or high chance) - 53.4% thought there was a low or no chance to get addicted when using hookah alone (46.8% felt there was a medium or high chance) - 82.5% felt hookah was socially acceptable among peers (17.5% felt not socially acceptable among peers) - 53.9% thought peers looked cool/very cool when using hookah (46.2% thought they looked not cool at all) |
| **Ahmed**  **2011 [**[**2**](#_ENREF_2)**]** | - Sampling frame: participants from the San Francisco Bay Area (N: not reported) - Sampling method: convenience sampling - Recruitment method: internet, email, flyers - Administration method: in person, administered by researcher | - Sample size calculation: no - Sampling type: non-probability sampling - Validity of tool: no validation reported. - Pilot testing done: not reported - Response rate: not reported | - Country: USA - Participants: waterpipe smokers the San Francisco Bay Area, mainly students from UC Berkeley (mean age: 22.9, 50% male, 58% of Asian origin, 26% White, 86% students) - Waterpipe smoking frequency:   Less than once a month- 50%  Once to five times a week- 50%   - Setting: San Francisco Bay Area, Jan to April 2008 - N sampled: not reported - N participated: 50 - N analyzed: 50 | - 88% of smokers considered hookah smoking to be harmful to health - What do you like most about hookah? Socialising (58%), flavour (28%), buzz (10%), energy (2%), the smoke (2%) - Plans to quit smoking hookah: don’t plan to (52%), at some point in life (28%), this month (2%), this week (2%), already quit (16%) - Factors leading to initiation: friends (84%), family (16%). - 52% believed hookah smoking is more harmful than cigarette smoking, 26% thought hookah was just as bad, 22% thought hookah was less harmful than cigarettes - 56% of respondents’ parents knew they smoked, of which 61% disapproved of their smoking, and 31% were indifferent |
| **Aljarrah**  **2009 [**[**3**](#_ENREF_3)**]** | - Sampling frame: all cafes that offered hookahs lounge in downtown San Diego, California and the surrounding suburb area (N: not reported) - Sampling method: convenience sampling - Recruitment method: in person - Administration method: in person, interviewer administered | - Sample size calculation: no - Sampling type: non-probability sampling - Validity of tool: self-developed tool, no validation reported - Pilot testing: not reported - Response rate: 99.6% | - Country: USA - Participants: waterpipe smokers at hookah lounges in downtown San Diego, California and the surrounding suburb area (mean age: 21.8, 57% male, 32.9% white, 22.4% Latino, 21.5% Middle Eastern, 11.0% Asian, 6.6% African American) - Cigarette smokers: 28.4% - Waterpipe smokers:   Daily- 13.5%  Weekly- 35.2%  Monthly- 24.4%  Every 6 months- 27%   - Setting: waterpipe smoking establishments, Aug to Sept 2008 - N sampled: 257 - N participated: 256 - N analyzed: 235 | - Perception of harm of waterpipe relative to cigarette: less (58.3%), more (30.4%) - Perception of waterpipe to be less harmful than cigarette: Whites, African Americans, and Middle Eastern ethnic groups more likely than Asians and slightly more than Latinos (p=0.03) - Hookah less harmful than cigarettes: 62.6% of males, 52.5% of females - Hookah more harmful than cigarettes: 9.9% of males, 13.1% of females - Hookah similar to cigarettes in harm: 27.5% of males, 34.3% of females |
| **Braun**  **2011 [**[**4**](#_ENREF_4)**]** | - Sampling frame: large Midwestern university (N: not reported) - Sampling method: simple random sampling - Recruitment method: email - Administration method: internet | - Sample size calculation: yes - Sampling type: probability sampling - Validity of tool: a standardized alcohol and drug survey which included 11 customized items related to hookah use was utilized. Face validity assessed by researchers. - Pilot testing: yes, done using students (N=19) as a convenience sample. Test-retest reliability reported with an average Pearson correlation coefficient of 0.76 - Response rate: 21.9% | - Country: USA - Participants: undergraduate students from a large Midwestern university (mean age: 23,1, 40% males , 77% white, 12% African American, 12% member of a fraternity or sorority - Waterpipe smoker:   Ever- 15.4%  Within past 30 days- 6%  Once a month- 42%   - Setting: university (internet), timing not reported - N sampled: 2,000 - N participated: 438 - N analyzed: 438 | - 98% reported that “close friend” introduced them to waterpipe smoking - 96% reported they primarily smoke waterpipe with friends - Reported reasons for waterpipe smoking: social gathering (29%), peer influence (27%), relaxation (25%) - 88% of smokers believed there were health hazards associated with hookah use - 98% of smokers believed they could quit at any time - Hookah smokers cited health effects of the hookah: respiratory effects (92%), cardiovascular effects (69%), cancer (69%) - 14% of hookah smokers were unsure what health effects were caused by hookah |
| **Chéron-Launay 2011 [**[**5**](#_ENREF_5)**]** | - Sampling frame: one high school in Paris (Nogent-sur-Marne) (N: not reported) - Sampling method: not reported - Recruitment: in person - Administration method: in person, self-administered | - Sample size calculation: no - Sampling type: not reported - Validity of tool: previously reported validated tool - Pilot testing: not reported - Response rate: not reported | - Country: France, Paris - Participants: students (mean age 15.4yrs, range 15-16yrs, 50.3% female) - Setting: high school, 2007 - N sampled: not reported - N participated: 300 - N analyzed: 300 | - La chichi apparaissait tres frequemment utilisee, y compris chez des jeunes se declarant non-fumeurs. |
| **Combrink 2009 [**[**6**](#_ENREF_6)**]** | - Sampling frame: secondary schools in Johannesburg (N: not reported) - Sampling method: purposive sampling - Recruitment method: in person - Administration method: self-administered | - Sample size calculation: no - Sampling type: non-probability sampling - Validity of tool: no validation reported - Pilot testing done: not reported - Response rate: not reported | - Country: Johannesburg, South Africa - Participants: students from high schools in Johannesburg (mean age: 16.3) - Waterpipe smoker: 60.9% - Cigarette smoker: 30.2% - Setting: high schools, timing not reported - N sampled: not reported - N participated: 202 - N analyzed: 202 | - Reason for smoking hookah: absence of alternative recreation (45.5%), peer pressure (13.8%), relaxation (27.6%), addiction (6.5%) - Among non-users, 92.4% would not consider waterpipe smoking in the future. - Among non-users, 39% had been subjected to peer pressure to smoke waterpipe - Perception of harm of waterpipe smoking relative to cigarettes: 32.2% of all respondents   - By non-users: less (10.1%), equal (64.6%), more (11.4%)  - By waterpipe smokers: less (52.8%), equal (42.3%), more (6.5%) |
| **Dillon 2010 [**[**7**](#_ENREF_7)**]** | - Sampling frame: African women leaders, African youth groups, and African social networks in Minnesota (N:148 to 342 for social networks, not reported for others) - Sampling method: interviews- expert sampling; focus groups- convenient sampling; survey- snowball sampling - Recruitment method: in person - Administration method: interviews- in person, interviewer administered; focus groups- in person, group discussion; survey- in person, self-administered with assistance for those who could not read proficiently | - Sample size calculation: no - Sampling type: non-probability - Validity of tool: self-developed, no validitation reported - Pilot testing done: not reported - Response rate: Surveys-65%. Not reported for interviews and focus groups. | - Country: USA - Participants: Interviews: community leaders. Focus groups: adolescents (mean age 16yrs, 56% Somali). Survey (67% Somali, mean age 35yrs) - Setting: Interviews: July – Aug 2008, unknown setting; Focus groups: Nov 2008, unknown setting. Survey Feb – Mar 2009, at community events, day cares, churches, homes. - N sampled: Interviews: 10; Focus groups: 29; Survey: 342 - N participated: Interviews: 10; Focus groups: 29; Survey: 223 - N analyzed: Interviews: 10; Focus groups: 29; Survey: 223 | - Many gaps in knowledge were seen particularly in regard to whether different types of tobacco products (e.g. menthol cigarettes, light cigarettes, shisha, smokeless tobacco) were safer than regular cigarettes. - 28% of survey respondents believed smoke from a shisha pipe is safer than smoke from cigarettes (10% believed it was not safer, 62% did not know) - The adolescents who participated in the focus groups demonstrated a general awareness of the negative impacts of smoking on health, appearance, and life. - In the interviews and focus groups, respondents expressed some confusion around whether shisha counts as tobacco, with one respondent stating that some people were trying to connect shisha to fruit because of the fruit-flavored varieties. - There was a common impression that shisha is safer than other tobacco products because of the flavor, smell, and presence of water. |
| **Eissenberg 2008 [**[**8**](#_ENREF_8)**]** | - Sampling frame: first-year university students enrolled in Virginia Commonwealth University, Introduction to Psychology Courses   (N = 1,194)   - Sampling Method: purposive sampling - Recruitment method : not reported - Administration method : internet, self-administered questionnaire | - Sample size calculation: no - Sampling type: non-probability sampling - Validity of tool: self developed tool, no validation reported - Pilot testing: not reported - Response rate: 62.3% | - Country: Virginia, USA - Participants: 1^st^ year university students, aged 18 and above (72% of students were between 18-19, 35% males, 57% whites, 23% African-Americans) - Waterpipe users:   Ever – 40.2%  Past year – 43.4%  Past 30 days – 20.4%   - Cigarettes:   Ever – 73.0%  Past year – 57.7%  Past 30 days – 41.5%   - Setting: university, Mar to May 2006 - N sampled: 1,194 - N participated: 744 - N analyzed:   744 | - Perceptions of waterpipe compared to regular tobacco products:   Never: never-users  Past: past 30-day users   - Perception that waterpipe is as harmful or more harmful compared to regular tobacco products: 63%   (Never: 81%, Past: 19%)   - Belief of getting addicted with a water pipe when using the product socially: Medium to high (57%), None to low (43%)   (Never: 66%, Past 36%)   - Belief that one looks cool to peers when they use a water pipe: Cool to very cool(36%), Not at all (64%)   (Never:23%, Past: 73%)   - Compared to past-30 day users, never-users of waterpipe are more likely to believe that waterpipe is as or more harmful than regular tobacco products , and that use of waterpipe socially increases the likelihood of becoming addicted - Compared to never users, past 30-day users are more likely to believe that they look “cool” to peers when they use a waterpipe |
| **Giuliani 2008 [**[**9**](#_ENREF_9)**]** | - Sampling frame: Somali community in Minnesota (N: not reported) - Sampling method: interviews: purposive sampling; focus groups: snowball sampling - Recruitment method: in person - Administration: in person, interviewer administered and group discussions | - Sample size calculation: no - Sampling type: non-probability sampling - Validity of tool:   - Key informant interviews, 14-item oral questionnaire from the Somali Tobacco Research Project, no validation reported  - Focus group discussions, 12 questions from a pre-established guide from the Somali Tobacco Research Project , no validation reported   - Pilot testing: not reported - Response rate: not reported | - Country: USA - Participants: - Key informant interviews- Somali community leaders and members and non-Somali professionals based on occupational connections to the Somali community in the areas of health, social services, and education (30% 40-49 yrs, 55% males, 35% college graduates) - Focus group discussions- youth of Somali ethnicity (46.7% males ) adults of Somali ethnicity (52.2% males, 79% less than a high school education) - Setting: Minnesota, fall 2006 and summer 2007 - N sampled: not reported - N participated: 20 (interviews), 91 (focus groups) - N analyzed: 20 (interviews), 91 (focus groups) | - Perception of smoking prevalence in Somali community: overall (M=50%), key informants (<1% to 70%), youth focus group participants (30-100%, M=64%), adult focus group participants (5-70%, M=37%) - Perception of smoking prevalence by smoking status: smokers (M=52%), non-smokers (M=47%) - Tobacco-use prevalence of female Somalis was universally perceived as being less than male Somalis, but members noted that estimated might be inaccurate as women tend to be closet smokers and hide their true smoking status - Perception of how a smoker can be identified:   - Physical appearance- smokers had darker lips, “looked dirtier”  - Health status- poorer health in general  - Attitude or character/ Financial- nervousness, impatience, irritability and anger, less respectful, less trustworthy, lazier, had misguided priorities, “were always begging”   - Reported reasons for tobacco-use initiation: influence of friends or peer pressure, fashionable or cool, stress or problems, family influences, fun or socialization, and loneliness - Regarding waterpipe smoking, respondents believed it was most popular with youth and utilized more frequently by women - Belief that fruity flavors and sweet smelling smoke are not seen as offensive and dirty since “cigarettes smell badly and it makes you feel nasty” - Belief that shisha is “fine and it calms you”, appears more feminine and not as embarrassing as cigarettes - Perception of harm of waterpipe smoking relative to cigarettes: opinion was divided, and several individuals felt there was no difference - Many who believed the waterpipe to be more benign felt it was not dangerous at all |
| **Giuliani 2010 [**[**9**](#_ENREF_9)**]** | - Sampling frame: Somali youth, grades 9-12, from Minnesota high schools (N: not reported) - Sampling method: school selection: probability-proportional-to; students selection: multistage sampling - Recruitment method: in person - Administration method: in person, interviewer administered | - Sample size calculation: no - Sampling type: probability sampling - Validity of tool: previously reported validated tool - Pilot testing: yes - Response rate: 86% | - Country: USA - Participants: Grades 9-12 Somali youth Minnesota (mean age 17.8, 48.3% females, but 3 gender values were missing, 80% born in Somalia) - Bidi and kretek smoking:   Ever- 9.3%  Current bidi smoking- 1.3%  Current kretek smoking- 1.0%   - Waterpipe smoking:   Ever- 3.8%  Current- 2.3%   - Cigarette smokers:   Ever- 12.8%  Current- 4.7%   - Setting: high schools, 2008 - N sampled: 350 - N participated: 302 - N analyzed: 302 | - Ever-users of tobacco were more likely to have close friends or live with someone who smokes cigarettes (22.2% vs 8.0%, p<0.01) or who use other various forms of tobacco (p<0.05) - Ever-users of tobacco were more likely to believe that using waterpipe/ shisha is less risky than smoking cigarettes (33.9% vs. 24.1%, p<0.01) |
| **Griffiths 2011 [**[**10**](#_ENREF_10)**]** | - Sampling frame: hookah establishments and a large Southeastern university (N: not reported) - Sampling method: snowball sampling - Recruitment method: in person - Administration method: in person, group discussion and phone | - Sample size calculation: no - Sampling type: non-probability sampling - Validity of tool: no validity reported - Pilot testing: not reported - Response rate: not reported | - Country: USA - Participants: college students who smoke waterpipe (mean age: 20.1, 50% male, 70% white, 40% junior, 25% sophomore, 15% freshman, 15% senior) - Cigarette smokers:   Ever- 0.1%  Current- 0.2%   - Setting: college campuses, hookah establishments, and via phone; timing not reported - N sampled: not reported - N participated: 20 - N analyzed: 20 | - Socialising with friends is a key driver for initital and continued hookah smoking - Consistent belief that hookah is more pleasurable, healthier and less risky than cigarettes - Belief that addictive effects unlikely because hookah is an occasional occurrence - Belief that safety of hookah evident from water filtering the smoke - Belief: Hookah lounges are safer environments for younger people…they stay out of trouble - Belief: Feeling of relaxation during and after smoking – head rush – a “nicotine rush” - Denial that hookah can be addictive - Belief that hookah does not contain the some chemicals as cigarettes - Motives:   - Peers strongly influenced smoking behavior  - Desire to smoke at home rather than at cafes because it’s cheaper and less time consuming  - Experimentation encouraged during group hookah sessions  - Hookah facilitates feeling part of a social group  - A hookah bar is a social compromise for younger consumers who cannot get into a bar due to age restrictions: an adult activity for underage people |
| **Jackson 2008 [**[**11**](#_ENREF_11)**]** | - Sampling frame: students of the University of Birmingham (N: not reported) - Sampling Method: cluster sampling - Recruitment method: in person - Administration method: in person, self-administered | - Sample size calculation: no - Sampling type: probability sampling - Validity of tool: self developed tool, no validation reported - Pilot testing: not reported - Response rate: not reported | - Country: UK - Participants: university students currently smoking waterpipe - Setting: waterpipe café near Birmingham University, timing not reported - N sampled: not reported - N participated: 21 of 75 waterpipe users (out of 937 who participated in the original survey) - N analyzed: 21 | - Belief waterpipe smoking is socially acceptable: 95% - Belief waterpipe smoking is bad for health: 91%; of these 68% believed waterpipe is less damaging than cigarettes |
| **Jamil 2010 [**[**12**](#_ENREF_12)**]** | - Sampling frame: White American adults residing in southeast Michigan (N: not reported) - Sampling method: purposive sampling - Recruitment method: in person - Administration method: in person, self-administered | - Sample size calculation: no - Sampling type: non-probability - Validity of tool: previously reported validated tool - Pilot testing: not reported - Response rate: > 98% (less than 2% refused) | - Country: USA - Participants: White American adults residing in Michigan (mean age 31, 61.2% female, 62.0% single, 67.9% non-student) - Current waterpipe smokers: 9.8% - Current cigarette smokers: 9.8% - Current waterpipe and cigarette smokers: 9% - Setting: community gatherings, June to Aug 2007 - N sampled: not reported - N participated: 315 - N analyzed: 245 | - Reasons for smoking hookah: socialize with family/friends (93.5%), taste (6.5%) - Intention to quit hookah: not at all (30.4%), in the future (69.5%) - Hookah is less harmful than cigarettes: yes (18.8%), no (20.8%), no response (60.4%) - Secondhand smoke from hookah is harmful: yes (20.8%), no (6.9%), don’t know (12.7%), no response (59.6%) |
| **Jamil 2011 [**[**13**](#_ENREF_13)**]** | - Sampling frame: Arab American adults residing in southeast Michigan (N: not reported) - Sampling method: purposive sampling - Recruitment method: flyers, mail - Administration method: in person, self-administered | - Sample size calculation: no - Sampling type: non-probability - Validity of tool: previously reported validated tool - Pilot testing: not reported - Response rate: not reported | - Country: USA - Participants: Arab American adults residing in Michigan - Waterpipe smokers:   Current- 29%  Ever- 8%   - Concurrent cigarette smokers:   Current- 53.8%  Ever- 5.1%   - Setting: The Arab American and Chaldean Council (ACC), Jan to Jun 2007 - N sampled: not reported - N participated: 904 - N analyzed: 801 | - Answering ‘yes’ to ‘is hookah smoking less harmful than cigarette smoking?’ 61.0% (current=70.7%, former=52.4%, never=44.3%) - Answering ‘yes’ to ‘is secondhand smoke from hookah harmful?’ 46.7% (current=36.4%, former=54.3%, never=66%) |
| **Lipkus 2011 [**[**14**](#_ENREF_14)**]** | - Sampling frame: university and college students from campuses in central North Carolina (N: not reported) - Sampling method: purposive sampling - Recruitment method: flyers, email - Administration method: internet | - Sample size calculation: no - Sampling type: non-probability - Validity of tool: no validity reported - Pilot testing: not reported - Response rate: not reported | - Country: USA - Participants: university and college students in central North Carolina who are waterpipe smokers (mean age: 20.4, 24.2% and 33.3% women in Study 1 and 2, respectively) - Setting: university (internet), Oct 2009 – Jan 2010, then follow up Feb – Mar 2010 - N sampled: study 1: 177; study 2: 153 - N participated: study 1: 108; study 2: 126 - N analyzed: study 1: 91 (follow up: 70); study 2: 112 | - Reasons for initiation of waterpipe (study 1 & study 2): curious about tobacco used (71.0% & 70.5%), like the way the waterpipe is crafted (20.0% & 6.2%), like the smell of waterpipe tobacco (48.9% & 46.4%), like the taste (61.1% and 54.5%), hookah bar/café opened nearby (41.1% & 43.8%), my friends use a waterpipe (82.2% & 83.9%) - Learned about waterpipe from friends: 95.6% & 94.6% - Smoke waterpipe as a social habit: yes absolutely (21.2% & 31.2%), yes probably (40.0% & 34.8%), yes maybe (21.1% & 23.2%), no (17.8% & 10.7%) - Participants who received information about harms and exposures compared to those who did not receive information:   - reported greater perceived personal risk of getting a serious smoking-related disease and expressed more worry about the physical consequences of waterpipe tobacco  - reported greater perceived personal risk and expressed more worry of becoming addicted  -reported a greater desire to quit |
| **Perusco 2007 [**[**15**](#_ENREF_15)**]**  **& Caroll 2008 [**[**16**](#_ENREF_16)**]** | - Sampling frame: Telephone listing “electronic white pages” - Sampling method: not reported - Recruitment method: phone - Administration method: phone | - Sample size calculation: yes - Sampling type: not reported - Validity of tool: self-developed, no validity reported - Pilot testing: not reported - Response rate: 70% | - Country: Australia - Participants: Arabic-speaking people in south-west Sydney, age range not reported (47.2% male) - Current waterpipe smokers: 11.4% - Current smokers: 26% - N sampled: 1,564 - N participated: 1,102 - N analyzed: 1,102 | - Smoking nargila/shisha is harmful to health: 81% (95% CI = 78-83%) - Smoke inhaled from nargila/shisha contains harmful chemicals: 73% (95% CI = 70-75%) - Respondents who did not smoke waterpipe were more likely to agree with above statements |
| **Primack 2008 [**[**17**](#_ENREF_17)**]** | - Sampling frame: University of Pittsburgh student registry e- email listing (N = 24,000) - Sampling Method: simple random sampling (computer generated) - Recruitment method: e-mail - Administration method : e-mail, self-administered | - Sample size calculation: yes - Sampling type: probability sampling - Validity of tool: Self developed tool modified from the American College Health Association (ACHA) National College Health Assessment (NCHA) - Pilot testing: not reported - Response rate: 18.33% | - Country: Pennsylvania, USA - Participants: university students (mean age 21, 34% males, 85% whites) - Waterpipe smokers   Ever – 41%  Past year – 30.6%  Past 30 days – 9.5%   - Cigarette smokers   Ever – 39.6%  Past 30 days – 21.5%   - Setting: university, timing not reported - N sampled: 3,600 - N participated: 660 - N analyzed: 647 | - Perception of harm of waterpipe relative to cigarette: less (33%), same (43%) more (24%) - Perception of addictiveness of waterpipe relative to smoking: less (52%), same (39%) more (9%) - Perception of peer acceptability of waterpipe: very acceptable (36%), somewhat/moderately acceptable (51%), not acceptable (12%) - Impression of popularity of waterpipe: very popular (30%), average (33%) not popular (37%) |
| **Richter 2006 [**[**18**](#_ENREF_18)**]** | - Quantitative (survey) and qualitative (focus groups) - Sampling frame: 16 focus groups obtained from focus group facility databases - Sampling method: purposive sampling - Recruitment method: phone, e-mail, plus advertising in campus newspapers - Administration method (survey):   - in person, self-administered | - Sample size calculation: no - Sampling type: non-probability sampling - Validity of tool: not reported - Pilot testing: not reported - Response rate: 58.55% | - Country: USA - Participants: young adult current smokers who tried or currently used non-traditional products, aged 18-22, males & females, [non-Hispanic white (52%), African-American (26.3%), Hispanics (22.6%)] - Waterpipe smokers - Ever – 4% - Setting: focus group facilities in Texas and Tennessee, Apr 2002 - N sampled: 234 - N participated: 137 - N analyzed: 137 | **Survey**:   - Shisha was rated as “safer than traditional cigarettes” (no quantitative results)   **Focus group**:   - Non-Hispanic white college students, Hispanic college students, African-American college students, and African-American not-in-college adults all perceived shisha to be “safer” than preferred cigarette variety - None of the groups surveyed thought that Shisha was “more harmful” than preferred cigarette variety |
| **Roskin 2009 [**[**19**](#_ENREF_19)**]** | - Sampling frame: Shisha cafes in England and Canada (N: not reported) - Sampling Method: snowball sampling - Recruitment method: snowball sampling - Administration method : in person, interviewer -administered | - Sample size calculation: no - Sampling type: non-probability sampling - Validity of tool: self-developed tool, no validation reported - Pilot testing: yes - Response rate: 40% | - Country: Birmingham, England and Toronto, Canada - Participants: patrons of waterpipe cafes, age and gender not reported - Setting: waterpipe cafes, early 2007 - N sampled: 30 - N participated: 12 - N analyzed: 12 | - Role of visits or links to Middle Eastern countries in initiation of waterpipe smoking - Seen as exotic and intimate - Social nature important in initiation and continued use - A mean to express heritage for Arabs. - Affordable relaxing novelty for Non Arabic students. - Relaxing appeal (fruit flavors, inhalation and exhalation of large quantities of smooth smoke) - More appealing than cigarettes - Feeling they wouldn't become addicted to it - Belief it is less harmful than cigarettes - Perception they weren't inhaling it despite inhaling for up to ten seconds at a time - Belief that the lack of media campaigns about water pipes implied they must be safer - Charcoal that burns the tobacco potentially harmful because it was a synthetic product - Belief the water has filtering properties. |
| **Smith 2007 [**[**20**](#_ENREF_20)**]** | - Sampling frame: E-mail address listing of students from Office of the Dean of Student Affairs, Johns Hopkins University   (N: not reported)   - Sampling method: not reported - Recruitment: method: e-mail - Administration method: Internet | - Sample size calculation: no - Sampling type: not reported - Validity of tool: self developed tool, no validity reported - Pilot testing: not reported - Response rate: 49.5% | - Country: Maryland, USA - Participants: college freshmen university students, aged above 18 - Waterpipe smokers:   Current: not specified  Ever: 12.7%   - Cigarette smokers:   Current: 19.5%  Ever: 26.0%   - Setting: university, spring 2004 - N sampled: 850 - N participated: 421 - N analyzed: 411 | - Perception of waterpipe harm relative to cigarettes: less (37%), equal or more (63%) |
| **Smith 2011 [**[**21**](#_ENREF_21)**]** | - Sampling frame: high school students in San Diego Country (N: 691) - Sampling method: purposive sampling - Recruitment method: in person - Administration method: in person, self-administered | - Sample size calculation: no - Sampling type: non-probability sampling - Validity of tool: no validation reported - Pilot testing: not reported - Response rate: 99.7% | - Country: USA - Participants: high school students from San Diego County (mean age: 17.1, 50.9% males, 33.5% Hispanic) - Waterpipe smokers:   Ever- 26.1%  Used in previous month- 10.9%  Recently smoked- 1%   - Cigarette smokers:   Ever- 37.7%  Used in previous month- 11.0%   - Setting: three schools in three suburban communities in San Diego county, 2010 - N sampled: 691 - N participated: 689 - N analyzed: 689 | - Most students first learned about waterpipe lounges from friends (50.3%) or saw a waterpipe lounge (20.9%) - 59.5% believe hookah is more socially acceptable than cigarettes - 46.3% think hookah is safer or less addictive than cigarettes. Reasons given were: no nicotine, less nicotine, not as addictive (33.1%); safer, less chemicals, cleaner (25.1%); cigarettes easier to get, more convenient, use more often (12.5%); it just seems like it, I was told that, I don’t know (9.5%); water filters the smoke, smoke is more filtered (6.1%); the tobacco/smoke is flavored (4.6%); hookah is more socially acceptable (3.0%); inhaling steam, water vapor, smoking air (2.7%); other (7.6%) - Belief that cigarettes are more harmful than cigars, smokeless tobacco, and waterpipe smoking, with waterpipe smoking being the least harmful (CI= 95%) - Hookah ever users were more likely to think hookah is socially acceptable (87.6% vs 46.7%, p<0.001) and safer/less addictive (78.2% vs 31.6%, p<0.001) than cigarettes, compared to non-users - Of the 180 ever hookah users, 93.1% are very confident they can quit anytime they want (5.1% confident). - Of the 180 ever hookah users, 29.3% do not plan to quit, 20.1% intend to quit in the distant future, 0.6% in the next 6 months, 5.2% in the next month - Of those who no longer smoke hookah, the reasons for stopping were: did not like it (21.6%), health reasons/hookah is bad for me (12.9%), started smoking cigarettes instead (4.1%), started using a different tobacco product (1.8%), not exposed to it (17.0%), other (7.6%), I still smoke hookah (42.1%) |
| **Smith-Simone 2008 [**[**8**](#_ENREF_8)**,** [**22**](#_ENREF_22)**,** [**23**](#_ENREF_23)**]** | - Sampling frame: E-mail address listing of students from Office of the Dean of Student Affairs, Johns Hopkins University (N: not reported) - Sampling method: not reported - Recruitment: method: e-mail - Administration method: Internet | - Sample size calculation: no - Sampling type: not reported - Validity of tool: self developed tool, no validity reported - Pilot testing: not reported - Response rate: 49.5% | - Country: USA - Participants: college freshmen university students, aged above 18 - Waterpipe smokers:   Current: not specified  Ever: 12.7%   - Cigarette smokers:   Current: 19.5%  Ever: 26.0%   - Setting: university, spring 2004 - N sampled: 850 - N participated: 421 - N analyzed: 411 | - Freshmen believed the following: - They were least likely to get addicted when using waterpipe socially compared to cigarettes or cigars - They were most likely to be influenced by their friends to use waterpipe in the next year than cigarettes and cigars - Waterpipe tobacco smoking was the most socially acceptable form of tobacco smoking among their peers - They perceived their peers to look coolest when using waterpipe instead of cigarettes and cigars - There was a high perceived likelihood of sickness waterpipe use alone in 240/402 respondents (59.7%). Of these 240, 81.7% were never waterpipe smokers, 10.4% ever smokers, 7.9% current smokers (p<0.001) - There was a high perceived likelihood of sickness waterpipe use socially in 213/402 (53%). Of these, never=84.5%, ever=9.4%, current=6.1% (p<0.001) - There was a high perceived likelihood of addiction waterpipe use alone in 275/402 (68.4%). Of these, never=81.1%, ever=8.7%, current=10.2% (p<0.001) - There was a high perceived likelihood of addiction waterpipe use socially in 222/402 (55.2%). Never=82.0%, ever=9.0%, current=9.0% (p<0.001) - There was a high perceived likelihood of benefit waterpipe use socially in 105/401 (26.2%). Never=63.8%, ever=11.4%, current=24.8% (p<0.01) |
| **Smith -Simone 2008 [**[**22**](#_ENREF_22)**]** | - Two sources:   1.) A waterpipe café in Richmond, Virginia  2.) Internet forum called Hookahforum.com  (N: not reported)   - Sampling Method: convenience sampling - Recruitment method: waterpipe café – word of mouth and flyer posted and circulated around the café; internet forum – forum thread posted by forum moderator - Administration method : waterpipe café – in person, self-administered | - Sample size calculation: no - Sampling type: non-probability sampling - Validity of tool: not reported - Pilot testing: not reported - Response rate: not reported | - Country: Virginia, USA - Participants: waterpipe café patrons and internet forum users, 86% of sample aged 18-24 (80.1% males, 85% whites) - Setting: waterpipe café and internet forum, May to Jul 2006 - N sampled: 201 (café patrons: 101, internet forum users: 100) - N participated = 201 - N analyzed = 201 | - Major reasons for waterpipe smoking (>50% of respondents): enjoy the taste (80%), good way of socializing with friends (80%), helps with relaxation (66%), enjoy smell (62%) - Confidence (moderate to very confident) could quit waterpipe smoking: 96% - No intention to quit: 68% - Belief they were not “hooked” or dependent on waterpipe: 87% - Belief that waterpipe is less harmful (67%), less addictive (79%), and has less nicotine (66%) than cigarettes - Belief that waterpipe will become more popular in the next 5 years: 78% - Perception of reduction of health risk with switching from cigarettes to water pipe (n= 197): none (17%), small (47%), moderate (29%), and large (7%) |
| **Sutfin 2011 [**[**24**](#_ENREF_24)**]** | - Sampling frame: seven public and one private universities in North Carolina (N: 5,000 to 40,000 each) - Sampling method: stratified random sampling - Recruitment method: email - Administration method: internet | - Sample size calculation: yes - Sampling type: probability sampling - Validity of tool: self-developed tool, no validation reported - Pilot testing: not reported - Response rate: 26.9% | - Country: USA - Participants: college students from universities in North Carolina (63% females, 80% white, 15% member of Greek organization) - Waterpipe smoker:   Current- 17.4%  Ever- 40.3%   - Cigarette smoker:   Current- 24.9%  Ever- 46.6%   - Setting: university, fall 2008 - N sampled: not reported - N participated: 3770 - N analyzed: 3770 | - Perception of harm of waterpipe smoking compared to cigarettes: more (17%), equal (50%), less (31%) - Among current (past 30 day) waterpipe users, 97% reported they could quit anytime, but only 53% planned to quit (of this 53%, 18% planned to quit in the next month, 2% in the next 6 months, 33% sometime in the future) |
| **Ward 2007 [**[**25**](#_ENREF_25)**]** | - Sampling frame: not reported (N: Not reported) - Sampling Method: convenience sampling - Recruitment method: flyers placed around universities, restaurants, cafes, where water pipe smoking occurs and in retail businesses where tobacco supplies; also phone recruitment - Administration method : in person, self-administered computerized survey | - Sample size calculation: no - Sampling type: non-probability sampling - Validity of tool: self-developed, no validity reported - Pilot testing: not reported - Response rate: not reported | - Country: USA - Participants: waterpipe users, majority aged 18-25 (75% males) - Setting: university, Mar to Nov 2005 - N sampled: not reported - N participated: 143 - N analyzed:   143 | - Did not consider themselves to be hooked on waterpipe: 91% - Were “very” confident they could quit at any time: 79% - Had no intentions to quit: 47% - Perception of harm of waterpipe relative to cigarette: less (67%), same (26%), more (7%) - Perception of addictiveness of waterpipe relative to cigarette: less (77%), same (20%), more (3%) - Perception of amount of nicotine in waterpipe smoke relative to cigarette smoke: less (64%), same (29%), more (8%) - Belief that switching from cigarette to waterpipe would reduce health risk by at least a small amount: 84% |
| **Weglicki 2008 [**[**26**](#_ENREF_26)**]** | - Sampling frame: two suburban Midwest high schools w/ enrollments > 45% Arab-American - Sampling Method: convenience sampling - Recruitment method : mail - Administration method: in person, self-administered, w/ researcher assistance | - Sample size calculation: no - Sampling type: non-probability sampling - Validity of tool: Previously reported validated tool (some items from YRBSS) - Pilot testing:   not reported   - Response rate: 99.9% | - Country: USA - Participants: Arab-American and non-Arab-American high school students (mean age: 15.6, 70% Arab-American, 30% non-Hispanic White) - ArA- Arab-American,   Non-ArA – non-Arab-American   - Cigarette smokers:   Ever –  ArA: 20.1%  Non-ArA: 39.3%  Current –  ArA: 6.9%  Non-ArA: 39.3%   - Waterpipe smokers:   Ever –  ArA: 38.0%  Non-ArA: 21.3%  Current –  ArA: 16.7%  Non-Ara: 11.3%   - Setting: two Midwestern high schools, 2004-2005 - N sampled: 2504 - N participated: 1874 - N analyzed: 1872 | - Perception of harm of waterpipe relative to cigarette: less (23%) equally (38%) more (39%) - Perception of waterpipe smoking being more harmful than cigarette smoking   - 24% of non Arab- American youth vs. 44% of Arab-American youth (P< 0.001)   - 37% of youth who experimented with water pipe vs. 31% who did not (P< 0.01) |
| **Middle-Eastern countries** | | | | |
| **Study** | **Methodology** | **Methodological qualities** | **Population and Setting** | **Results** |
| **Afifi 2009 [**[**27**](#_ENREF_27)**]** | - Sampling frame: three urban neighborhoods in Beirut (N: Nabaa 9,000-12,000; Burj El Barajneh 14,000-18,000; El Sellom 100,000-120,000) - Sampling Method: the neighbourhoods were divided into blocks. From a sample of those blocks, a sample of households was then selected from each block. - Recruitment method: in person - Administration method: in person, interviewer administered | - Sample size calculation: yes (done for prevalence) - Sampling type: probability - Validity of tool: no validation reported - Pilot testing: not reported - Response rate: 71%, 85.6% and 96.4% | - Country: Lebanon - Participants: adolescents from three urban disadvantaged neighborhoods of Beirut (mean age: 17, male to female ratio about 1:1) - Narghile smoking:   More than 50% ever tried narghile; of these 39% continued to smoke   - Setting: neighborhoods in Beirut, late spring 2002 (initial household suvey) and winter and early spring of 2003 (population subgroups surveys) - N sampled: not reported - N participated: 1294 - N analyzed: 1294 | - Perception that narghile is more harmful than cigarettes: 57.4% - Males who were not enrolled in an educational institution, those who stated they were not religious, those who had high stress, those who smoked cigarettes and those who rated their health as poor or average were more likely to have continued to smoke narghile after the first puff . - With respect to social influences, adolescents who had friends who smoke and whose friends encouraged them to smoke were more likely to continue than those who did not. - Narghile seen as more harmful than cigarette smoking by 20.2% of narghile smokers |
| **Al -Dabbagh 2005 [**[**28**](#_ENREF_28)**]** | - Sampling Frame: 20 water pipe cafes in 3 Iraqi cities: Mosul, Baghdad, and Senjar   (N: Not reported)   - Sampling Method: not reported - Recruitment method: in person - Administration method: in person, interviewer administered. | - Sample size calculation: no - Sampling type: not reported. - Validity of tool: not reported - Pilot Testing: not reported - Response Rate: 100%. | - Country: Iraq - Participants: People present at the water pipe café (mean age 31.6% for smokers, 100% males) - Setting: waterpipe cafes, timing not reported - N sampled: 200 - N participated: 200 - N analyzed: 200 | - Preferred Nargila smoking than cigarettes, because they believe it is less harmful (due to passage of smoke through water) and more enjoyable: 90% - Unwilling to quit smoking Nargilas: about 50% - Reasons of using water pipe:   - Entertainment: 70%   - Addiction: 13%   - A way to quit smoking cigarettes: 9% |
| **Allam 2007 [**[**29**](#_ENREF_29)**]** | - Sampling frame: Ain Shams University Students’ Unions (ASU-SU; N: not reported) - Sampling method: simple random sampling - Recruitment method: in person - Administration method: in person, interviewer administered | - Sample size calculation: no - Sampling type: probability sampling - Validity of tool: no validation reported - Pilot testing: yes - Response rate: not reported | - Country: Egypt - Participants: male students from ASU-SU - Smokers (cigarette and shisha): 31.5% - Setting: university student union, March to April 2005 - N sampled: not reported - N participated: 108 - N analyzed: 108 | - Among the smoking students, 44.1% felt that quitting smoking is not possible at all. - No intention to smoke after one year (72.2%) - No intention to smoke after five years (44.4%) - 84.3% knew shisha is hazardous - 55.6% believed shisha smoking is more hazardous that cigarettes |
| **Almerie 2008 [**[**30**](#_ENREF_30)**]** | - Sampling frame: registrar of list of medical students at Damascus University School of Medicine (N: 1173) - Sampling method: 1^st^ and 5^th^ year students were randomly selected from the registrar - Recruitment method: in person - Administration method: in person, self-administered | - Sample size calculation: yes - Sampling type: probability - Validity of tool: previously reported validated tool - Pilot testing: not reported - Response rate: 93.1% | - Country: Syria - Participants: 1^st^ and 5^th^ year registered medical students at the Damascus University School of Medicine (mean age: 20.5, 51.4% first year) - Tobacco smokers: 10.9% (15.8% male, 3.3% female) - Waterpipe smokers: 23.5% (30.3% male, 13.4% female) - Both tobacco and waterpipe smokers: 27.1% (36.0% male, 13.6% female) - Setting: university, 2006-2007 - N sampled: 612 - N participated: 570 - N analyzed: 570 | - Almost half the respondents think that male smokers look less attractive, compared to 80% who think that female smokers look less attractive. - People who thought waterpipe smoking is worse than cigarette smoking: cigarette smokers (84.8%), cigarette non-smokers (74.2%), waterpipe smokers (76.4%), waterpipe non-smokers (74.3%) - 67.3% of waterpipe smokers, and 62.9% of non-waterpipe smokers, think smoking waterpipe is religiously unacceptable - 56.7% of waterpipe smokers, and 52.9% of non-waterpipe smokers, think quitting smoking is difficult |
| **Anjum 2008 [**[**31**](#_ENREF_31)**]** | - Sampling frame: listing of schools in Directorate of Schools and Colleges (N: not reported) - Sampling method: pretest used convenience (for schools), multistage (for participants); post-test used purposive sampling - Recruitment method: in person - Administration method: in person, self-administered survey with interactive health sessions | - Sample size calculation: yes - Sampling type: non-probability - Validity of tool: no validation reported - Pilot testing: not reported - Response rate: not reported | - Country: Pakistan - Pre-Test   - Participants: adolescents from high, middle, and low socioeconomic groups studying at different educational institutions in Karachi, Pakistan (mean age: 15.3, 57% males)  - Waterpipe Smokers:  Ever- 27%  Current- 17%   - Post-Test   - Participants: adolescents from high and middle socioeconomic groups (mean age: 15.2, 37% males)  - Waterpipe Smokers:  Ever- 24%  Current- 14%   - Setting: high schools, timing not reported - N sampled: not reported - N participated: 646 pre-test, 250 post-test - N analyzed: 646 pre-test, 250 post-test | - Of current waterpipe smokers in the pretest, 35/109 (32.1%) wanted to quit waterpipe smoking, and 30/109 (27.5%) had made an attempt to quit. In the post test, 18/34 (52.9%) of current smokers wanted to quit, and 9/34 (26.5%) had made an attempt to. - Of the non-smokers, 7% wanted to try waterpipe smoking soon in the pre-test, and 8% wanted to try it in the post-test. - Upon asking all respondents who: - Believed waterpipe is addictive: 54% in pre-test, 68% in post-test - Believed waterpipe is more addictive than cigarettes: 11% in pre-test, 32% in post-test - Believed waterpipe is more harmful compared to cigarette: 16% in pretest, 45% in post test - Perceived waterpipe smokers to look cool: 24% in pretest, 24% in post test - Believed waterpipe smokers have more friends: 33% in pretest, 32% in post test - Believed waterpipe smoking is more socially acceptable compared to cigarette: 58% in pretest, 80% in post test - Believed waterpipe smoking is their cultural heritage: 29% in pretest, 58% in post test - Believed girls are more comfortable in smoking waterpipe compared to cigarettes: 66% in pretest, 79% in post test - Obliged to smoke if friends are: 17% & 21% - Cafes playing an important role in promotion of waterpipe: 89% & 92% - Waterpipe makes boys more attractive: 19% & 20% - Waterpipe makes girls more attractive: 17% & 18% - Belief in health hazards (pre and post-test): cardiovascular (24% & 10%), respiratory (70% & 72%), cancer (41% & 37%), other bodily effects (18% & 23%), oral infections (12% & 17%), none (9% & 6%) - Belief in associated diseases (pre and post-test): bladder cancer (19% & 33%), bronchitis (50% & 56%), oesophageal cancer (29% & 35%), depression (25% & 23%), high blood pressure (31% and 16%), Parkinson’s disease (5% & 6%), ulcer (19% & 18%), lip cancer (35% & 61%), impaired pulmonary function (26% & 24%), infections (35% & 34%), infertility (10% & 38%) |
| **Aydin 2011**  **[**[**32**](#_ENREF_32)**]** | - Sampling frame: not reported (N: not reported) - Sampling method: simple random - Recruitment method: not reported - Administration method: not reported | - Sample size calculation: 600 - Sampling type: probability sampling - Validity of tool: no validation reported - Pilot testing done: not reported - Response rate: not reported | - Country: Turkey - Participants: teachers from schools in Turkey (male to female ratio 1.04, mean age 34.2) - Current smokers: 27.6% - Setting: school, timing not reported - N sampled: not reported - N participated: 633 - N analyzed: 633 | - 30.5% of teachers think that waterpipe, light cigarette, tobacco pipe and Maras powder is less harmful than cigarette smoking.  \|  \| \| --- \| |
| **Azab 2010 [**[**33**](#_ENREF_33)**]** | - Sampling frame: four universities in Jordan (N: not reported) - Sampling method: two-stage cluster sampling and systematic random - Recruitment method: in person - Administration method: In person, interviewer administered | - Sample size calculation: no - Sampling type: probability sampling - Validity of tool: previously validated instruments - Pilot testing done: not reported - Response rate: 75.1% | - Country: Jordan - Participants: undergraduate students from universities in Jordan (mean age 21.7, 51.8% males, 92.8% Jordanian, 28.3% fourth year, 92.0% single, 36.3% arts, 30.4% general sciences, 24.8% medicine) - Waterpipe smokers:   Ever- 61.1%  Monthly- 42.7%   - Cigarette smokers:   Ever- 56.6%   - Setting: university, March to July 2008 - N sampled: 735 - N participated: 552 - N analyzed: 548 | - Perception of harm of waterpipe smoking relative to cigarettes: more (62.2%), equal (28.0%), less (9.8%) - Perception of addiction to waterpipe smoking relative to cigarettes: more (13.2%), equal (32.2%), less (54.6%) - Although those believing that cigarettes were more harmful than water pipe were more commonly waterpipe smokers, the relationship was only significant for those who smoked waterpipes at least monthly (p<.001) and not for ever-use (p<.09) |
| **Chaaya 2004 [**[**34**](#_ENREF_34)**]** | - Sampling frame: network of the reproductive health program (N=1863) - Sampling method: stratified cluster sampling - Recruitment method: in person - Administration method: in person, interviewer administered | - Sample size calculation: no - Sampling type: probability sampling - Validity of tool: self developed tool, validation reported (content validity) - Pilot testing done: yes - Response rate: 100% | - Country: Lebanon - Participants: pregnant women (mean age 27.3; 14.1% persistent cigarette smokers; 3.6% persistent arguileh smokers) - Setting: women with prenatal visits to selected clinics, Feb 2003 - N sampled: 909 - N participated: 864 - N analyzed: 864 | - Support smoking ban, for < 18 years of age, on:   - Arguileh: 75.6%   - Cigarette: 81.6% - Beliefs that arguileh and cigarettes respectively:   - Contains addictive substances: 45.2%, 68.7%   - Produces harmful gases: 39.4%, 53.3%   - Contains carcinogens: 41.3%, 69%   - Affects the fetus: 73.9%, 78.4%   - Affects the newborn: 70.6%, 76.8% |
| **Dar- Odeh 2010 [**[**35**](#_ENREF_35)**]** | - Sampling frame: registrars from three public Jordanian universities- Jordan University of Science and Technology (JUST), University of Jordan (UJ), and Mu’tah University (MU) (N: 20,409; 38,690; and 17,000 respectively) - Sampling method: simple random sampling - Recruitment method: in person - Administration method: in person, self administered, with assistance where required | - Sample size calculation: no - Sampling type: probability sampling - Validity of tool: no validation reported - Pilot testing done: not reported - Resposne rate: not reported | - Country: Jordan - Participants: students from three public Jordanian universities (ages 16-26, 60% male, 36.8% smokers) - Setting: university, Dec 2008 - N sampled: not reported - N participated: 1454 - N analyzed: 1454 (741 males and 712 females; one student failed to state gender) | - 143 (9.8%) students thought smoking narghile is more harmful than cigarette smoking while 1165 (80.1%) thought it was not. - The most frequently stated harmful effects of narghile were: respiratory disease (540, 37.1%), cancer (503, 34.6%), cardiovascular disease (291, 20.0%) and mouth disease (85, 5.8%). |
| **Erbaydar 2010 [**[**36**](#_ENREF_36)**]** | - Sampling frame: nine narghile cafés in Ankara, Turkey (N: not reported) - Sampling method: regular narghile smokers were approached in nine narghile cafes - Recruitment method: in person - Administration method: in person, interviewer administered | - Sample size: not calculated - Sampling type: not reported - Validity of tool: no validation reported - Pilot testing done: not reported - Response rate: not reported | - Country: Turkey - Participants: narghile smokers in cafes (mean age: 22.5, 76.7% males, 63.0% students) - Setting: narghile cafes, 2004-2005 - N sampled: not reported - N participated: 460 - N analyzed: 460 | - The most common reasons for starting narghile smoking: peer influence (38.4%), curiosity (18.4%), and influence of family members that smoke narghile (11.8%), relaxation (9.1%), enjoy taste (7.3%), influence of social environment (4.0%), imitation (3.8%), instead of cigarette (1.8%), others (6.0%) - 47.8% had family members who smoked narghile - 50.1% did not believe narghile smoking is addictive (yes=34.6%, no idea=15.3%) - 23.3% believed communicable disease could be spread by shisha (did not believe=35.7%, no idea=41.0%) - On a scale where 0=not harmful and 10= very hazardous, 74.9% of respondents rated cigarette’s health hazard between 8.1 and 10 compared to 32.4% for narghile. |
| **Ghafouri 2011 [**[**37**](#_ENREF_37)**]** | - Sampling frame: a health sciences university in Iran   (N: 2250)   - Sampling Method: not reported - Recruitment method: in person - Administration method: in person, self-administered | - Sample size calculation: no - Sampling type: not reported - Validity of tool: previously validated tools - Pilot testing: yes - Response rate: 96.5% | - Country: Iran - Participants: first year graduate students (mean age 22 years, 53% female) - Setting: university, 2 weeks over Dec 2007 - N sampled: 371 - N participated: 358 - N analyzed: 296 | - Motivating factors to smoke waterpipe among current waterpipe smokers: fun and social aspect (75.5%), curiosity (11.1%), association with smokers (24.3%), habit (31.1%), addiction (17.2%) - Motivating factors to smoke waterpipe among ever waterpipe smokers: fun and social aspects (65.3%), curiosity (42.3%), association with smokers (26.8%) - Comparing perception of waterpipe smoking by current vs occasional waterpipe smokers: healthiest way to use tobacco (40.6% vs 11.1%) , makes one look attractive (8.8% vs 4.4%), dangerous to health (67.7% vs 73.3%), relaxing (62.5% vs 26.2%), gives them energy (48.5% vs 11.4%), has a pleasant taste and smell (73.5% vs 51.1%), I smoke because it’s a way of my culture (58.8% vs 34.1%), addictive (20.6% vs 22.2%) - While a large majority (73.3% and 67.7%) of both groups felt that waterpipe smoking was dangerous for their health, paradoxically only 22.2% and 20.6% in the occasional and frequent group, respectively, felt that it was addicting. - A greater number of frequent waterpipe smokers perceived that waterpipe smoking was the healthiest way to use tobacco than occasional waterpipe users. |
| **Hammal 2008 [**[**38**](#_ENREF_38)**]** | - Qualitative study - Sampling frame: not reported - Sampling method: not reported. - Recruitment method: newspaper advertisements and word-of-mouth - Administration method: in person, interviewer administered | - Sample size calculation: no - Sampling type:   not reported   - Validity of tool: self developed tool, no validation reported   (they followed an interview guide that covered smoking initiation, current patterns of use, dependence, perceptions about health effects, interest in quitting, previous quitting experiences)   - Pilot testing: not reported - Response rate: not reported | - Country: Aleppo, Syria - Participants: Adult smokers of cigarette and waterpipe from different professions, aged 18 and above (81.25% males) - Setting: community - Study: community, timing not reported - N sampled: not reported - N participated: 32 (16 cigarette and 16 narghile smokers) - N analyzed: 32 | - Narghile smokers found that narghile was a pleasurable social experience embedded in cultural rituals. In contrast, cigarette smokers saw their cigarette smoking as a mundane, oppressive, personal addiction - Narghile smokers found that smoking narghile fostered a sense of togetherness and cultural identity - Unlike cigarette smokers who felt stigmatized, narghile smokers generally felt that smoking narghile was socially accepted - Narghile smokers believed that smoking narghile was relatively harmless to themselves or to others - Narghile smokers used narghile for entertainment, leisure, and escape - Frequent narghile smokers confessed that they felt addicted in much the same way as cigarette smokers - Both cigarette smokers and narghile smokers viewed quitting as a matter of will and conviction - Most cigarette smokers had tried to quit, compared to very few water pipe smokers had ever tried to quit, and most were not interested in quitting - Family’s attitude toward tobacco use:   - Positive : 31.25%   - Neutral: 31.25%   - Negative: 37.5% |
| **Israel 2003 [**[**39**](#_ENREF_39)**]** | - Sampling frame: 9 different shisha cafes in Cairo, Egypt   (N: not reported)   - Sampling method: not reported - Recruitment method: in person - Administration method: in person, interviewer administered | - Sample size calculation: No - Sampling type: not reported - Validity of tool: not reported - Pilot testing: not reported - Response rate:   100% | - Country: Cairo, Egypt - Participants: shisha café patrons aged 18 and above (mean 33.2 years, 99% males) - Setting: waterpipe cafes, timing not reported - N sampled: 300 - N participated: 300 - N analyzed: 297   Note: females left out because only 3 surveyed | - Top reasons for visiting Shisha cafés:   - Spending time w/ friends: 77%  - Family safety: 71%  - Shisha not easy to prepare at  home: 70%   - 69% preferred Shisha smoking to cigarette smoking - Top reasons for preferring Shisha over cigarettes:   - Done out of habit: 27%   - Decreasing smoking hours: 24%   - Less harmful: 21% - Most respondents were aware of the health effects of Shisha:   - Transmits infections: 92%   - Yellowish teeth: 89%   - Bronchial asthma: 85%   - Lung cancer: 84%   - Heart disease: 81%   - Hypertension: 57% - Younger age groups (18-25) were significantly more aware of the health effects - Felt they could stop smoking whenever they want: 78% - Wanted to quit: 62% - Attempted to quit smoking for even one day the last year: 52% - Tried to quit smoking during the past year (>2 times): 19% - Thought that Shisha was harmful: - Strongly disapprove their sons (84.2%) or daughters (97.6%) to smoke - Would advise son/daughter to quit if they were found smoking (81.6%) |
| **Jawaid 2008 [**[**40**](#_ENREF_40)**]** | - Sampling frame: list of all public and private universities from Higher Education Committee (HEC) (N: not reported) - Sampling Method: multistage stratified sampling (including random then systematic sampling) - Recruitment method : in person - Administration method : in person, self-administered | - Sample size calculation: yes - Sampling type: probability sampling - Validity of tool: Self developed tool based on previously developed tools , no validation reported - Pilot testing: yes - Response rate: 92% | - Country: Karachi, Pakistan - Participants: university students fluent in English (mean age: 21, 59.6% males) - Waterpipe smokers:   Ever – 53.6%   - Setting: university, Mar 2006 to Mar 2007 - N sampled: 487 - N participated: 450 - N analyzed: 450 | - Most common reasons for starting shisha smoking:   - Curiosity: 61%   - Pleasure-seeking : 47%   - Peer pressure: 23% - Identified flavor as the most likeable attribute of the waterpipe: 71% - Considered themselves addicted to waterpipe: 32% - Reported that parents approved of waterpipe smoking: 79%; and cigarette smoking: 21.1% - Unable to identify a single harmful effect of waterpipe smoking: 18% - The vast majority of participants viewed cigarette smoking as more hazardous than shisha smoking - Belief water pipe contains significant amounts of tobacco (56%); has side effects other than those of cigarettes (23%); can cause cancer (53.3%), respiratory problems (73% cardiovascular impairments (37%), hematological disease (11%); is hazardous during pregnancy (29%); is not associated with any hazards (18%) - Beliefs water pipe has an efficient filtration mechanism (59%), detoxifies smoke through its fruit flavor (7%), contains less nicotine than cigarettes (47%); less frequently of use limits its side of effects (54%); waterpipe is less irritating to the respiratory tract than cigarettes ( 25%) |
| **Kelishadi 2007 [**[**41**](#_ENREF_41)**]** | - Sampling frame: population of youths aged 12-20 in Isfahan, Iran   (N: not reported)   - Sampling Method: convenience sampling - Recruitment method: not reported - Administration method: in person, self-administered questionnaire | - Sample size calculation: yes - Sampling type: non-probability sampling - Validity of tool: Self developed tool, content validity established, reliability tested with Cronbach alpha coefficient of 0.72 - Pilot testing: yes - Response rate: 95.7% | - Country: Isfahan, Iran - Participants: youngsters who were smoking in parks within different parts of the city and non-smokers, aged 12-20 (75.6% males) - Setting: community, 2004 - N sampled: 441 - N participated:   427  Smokers – 210  Non-smokers - 217   - N analyzed: 427 | - 97% of smoking girls and 92% of smoking boys revealed that they used waterpipe as a means of entertainment, hospitability, and as a symbol of fashion/fad |
| **Labib 2007 [**[**42**](#_ENREF_42)**]** | - Sampling frame: 9 waterpipe cafés near two universities (N: not reported) - Sampling method: not reported - Recruitment : method: in person - Administration method: in person, self-administered | - Sample size calculation: no - Sampling type: not reported - Validity of tool: self developed tool, no validation reported - Pilot testing: not reported - Response rate: 100% | - Country: Cairo, Egypt - Participants: female medical students from Cairo University, and undergraduate science students (mean age: 21) - Smoked waterpipe only:   37.8%; smoked cigarettes only: 27%; smoked both: 35.2%   - Setting: waterpipe cafes in the vicinity of the two universities, 2004 - N sampled: 196 - N participated: 196 - N analyzed: 196 | - 74% of waterpipe smokers preferred this method because they believe it to be less harmful than smoking cigarettes - Reasons for choosing water pipe over cigarettes:   - It is fashionable: 20%   - It is less harmful: 19%   - To be with my friends: 18% |
| **Makhoul 2009 [**[**43**](#_ENREF_43)**]** | - Quantitative (survey) and qualitative (focus group) - Sampling frame: households from three communities in Beirut- Borj Barajneh Palestinian Refugee Camp, Hay el Sullum, and Nabaa (N: 14,000 to 18,000) - Sampling method: probability proportional to size sampling - Recruitment method: in person - Administration method: in person, interviewer administered | - Sample size calculation: no - Sampling type: probability sampling - Validity of tool: no validation reported - Pilot testing done: yes - Response rate: 84.8% (survey of adolescents); not reported for survey of households or focus groups | - Country: Lebanon - Survey Portion of Study   - Participants: adolescents from three urban communities in Beirut  - Cigarette smokers- 8.3%  - Argileh smokers- 22.4%  - Setting: urban communities, 2003  - N sampled: 1526  - N participated: 1,294  - N analyzed: 1,294   - Focus Group:   - Participants: adolescents from Borj Barajneh camp  - Setting: refugee camp, timing not reported  - N sampled: not reported  - N participated: 482  - N analyzed: 482 | - Participants in the focus group felt some behaviors were underreported in the questionnaire results: in the case of cigarette and argileh smoking (8.3% and 22.4%, respectively), the participants affirmed that in reality many more adolescents smoke and begin at a very early age. - Interviewees in the survey ay have feared admitting cigarette smoking because of social undesirability and parental disapproval. - Participants believed far more boys than girls smoke cigarettes, which is concurrent with survey findings. - The boys participating in the focus group were aware of the negative health effects of smoking and nevertheless continued to smoke. - Those who smoke and use drugs are considered to be less healthy than others who do not. - Young people use drugs to “forget problems,” as a group of 13-19 year old boys mentioned, especially when adolescents have nobody to listen to at home at or at school. - Girls mentioned that smoking is linked to the culture of the camp society and consequently their homes. - Boys choose to smoke because they think it is an expression of their manhood, and smoking argileh is spreading fast due to its social acceptance as compared to smoking cigarettes. - Some families might even encourage their children to smoke argileh at home in social gatherings. |
| **Maziak 2004 [**[**44**](#_ENREF_44)**]** | - Sampling frame: participants from two previous studies (Aleppo university students - student registry, and waterpipe café patrons)   (N: not reported)   - Sampling Method: simple random sampling and convenience sampling - Recruitment method : in person - Administration method :   in person, interviewer administered | - Sample size calculation: no - Sampling type: non-probability sampling - Validity of tool: self-developed tool, validation reported   (from standardized questionnaires used in international settings)   - Pilot testing: yes - Response rate:   98.8%  Students;  95.4%  waterpipe;  97% overall  response rate | - Country: Aleppo, Syria - Participants: university students and waterpipe smokers, aged 18-30 for students and aged 18-68 for waterpipe smokers (mean: 24.4) - Setting: university and waterpipe cafes   2003   - N sampled: 881 - N participated: 855 - N analyzed: 855 | - Smoking by women was associated with less positive perceptions than by men - Smoking cigarettes was associated with less positive perceptions than narghile - Cigarette smoking by women was least associated with positive attributes - Perception of any type of smoking was generally more favorable among city residents, better-off participants, and Christians, while perception for narghile smoking was favorable among older and married participants - Smokers generally associated more positive attributes to smoking regardless of gender, but were more positive about the method they use (i.e., cigarette smokers are more appreciative of cigarette smoking and vice versa) |
| **Maziak 2004 [**[**45**](#_ENREF_45)**]** | - Sampling frame: studentship at Aleppo University in 2001 (N = 43,000); sampling frames w/in each of the 4 men’s and 4 women’s dorms - Sampling Method: stratified random sampling - Recruitment method:   in person   - Administration method: in person, interviewer administered | - Sample size calculation: no - Sampling type: probability sampling - Validity of tool:   self developed tool, validation reported (tool based on relevant instruments used for the assessment of cigarette smoking [IUTLD, WHO], and from previous questionnaires used in Syria)   - Pilot testing: yes - Response rate: 98.8% | - Country: Aleppo, Syria - Participants: university students (mean age 21.8 years, 47.4% males) - Waterpipe smokers:   Ever – 36.8%  Current – 24.7%   - Setting: university, 2003 - N sampled:   not reported   - N participated: 587 - N analyzed : 587 | - Most common positive perceptions of narghile were its smell (26%) and taste (9%). - Most negative perceptions of narghile were the smoke and pollution (25%), and perceived adverse health effects (19%). - Belief that narghile, compared to cigarettes, is more harmful:50%; less harmful: 30%; equally harmful: 19 % - Top reasons for believing narghile is more harmful: Every nafas equals no. of cigirattes (19%), more inhalation of smoke (18%), tobacco used in narghile is more harmful (16%) - Belief that narghile, compared to cigarettes, is more addictive: 4%; less addictive: 81%; equally addictive: 15% - Family attitudes regarding tobacco use by younger members were more permissive about narghile compared with cigarettes, and, about females smoking narghile than males doing so |
| **Maziak 2004 [**[**46**](#_ENREF_46)**]**  **Ward 2005 [**[**47**](#_ENREF_47)**]**  **Asfar 2005 [**[**48**](#_ENREF_48)**]** | - Sampling frame: list of all cafés/restaurants serving narghile in Aleppo, Syria from Dept of Tourism(N= 2.5 million) - Sampling Method: multistage random sampling - Recruitment method: not reported - Administration method: in person, interviewer administered | - Sample size calculation: no - Sampling type: probability sampling - Validity of tool:   previously  reported  validated tool   - Pilot testing: yes - Response rate: 95.3% | - Country: Aleppo, Syria - Participants: adult narghile users, 18 yrs and above (mean 30.1, 60.1% males) - Setting: cafes and restaurants serving narghiles in Aleppo, Syria, 2003 - N sampled: 280 - N participated: 268 - N analyzed: 268 | - Frequency of narghile use was strongly correlated w/ participant’s subjective judgment of how hooked they are on narghile. - How hooked on water pipe:   - Not hooked: 44.7%   - Somehow hooked: 40.2%   - Very hooked: 14.5% - Belief that they could quit (among users who thought they were “very hooked”): 49% - Interest in quitting: 28% - Among those interested in quitting:   - health concerns as the primary reason for quitting: 89%   - did not expect any particular challenges: 62%   - Challenges cited: - Boredom and free time: 24% - Socializing with friends who were smoking: 21% - Coping with the habit or addictiveness: 17% - Among waterpipe smokers who also smoked cigarettes, belief that quitting waterpipe was easier than quitting cigarettes: 80% - Interest in quitting cigarettes was more common than interest in quitting waterpipe: 63% vs. 25% - Belief:   - Able to quit waterpipe anytime: 87%   - Waterpipe is more harmful than cigarette: 25%   - Water pipe is more addictive than cigarette: 7%   - Waterpipe is as difficult or more difficult to quit than cigarettes: 19%   - Families disapproves of water pipe smoking: 26% - Families were more tolerant of women who smoke using waterpipe compared to men - Women seemed to be drawn to the habit despite the taboo regarding women smoking - Established waterpipe smokers (café) were more smoking-method oriented, more hooked on smoking, less willing to quit, and lees likely to foresee challenges to quitting vs. nonsmokers (university students) - Belief about the addictive effects of water pipe compared to cigarettes:   - Cigarettes are more addictive: 84.1%;   - Equally addictive: 9.6%   - Water pipe is more addictive: 5.9% - Belief about harmful effects of water pipe compared to cigarettes:   - Cigarettes are more harmful: 44%   - Equally harmful: 16.9% - Water pipe is more harmful: 38.4% |
| **Mohammed**  **2010 [**[**33**](#_ENREF_33)**,** [**49**](#_ENREF_49)**]** | - Sampling frame: university, organized meetings, and various settings in Kuwait (N: not reported) - Sampling method: purposive sampling - Recruitment method: in person - Administration method; in person, self-administered | - Sample size calculation: no - Sampling type: non-probability - Validity of tool: no validation reported - Pilot testing done: yes - Response rate: 82% | - Country: Kuwait - Participants: individuals from various settings in Kuwait (52.9% male, 11.6% first year students at Kuwait University, 17.3% students from the Kuwait interior police academy, 53.2% unskilled manual workers, and 17.9% professional workers) - Waterpipe smokers: 44.6% - Cigarette smokers: 12.4% - Both waterpipe and cigarette smokers: 8.0% - Setting: various settings in Kuwait, timing not reported - N sampled: 3624 - N participated: 2972 - N analyzed: 2972 | - 50.6% of the waterpipe only smokers reported that all or most of their friends smoked waterpipes, compared with 42.4% of the cigarette only smokers and 65.7% of those who smoked both waterpipe and cigarettes. - 47.5% of the waterpipe smokers said it was friends who first encouraged them to try the waterpipe. - 57.7% of the waterpipe smokers and 52.6% of those who smoked both waterpipe and cigarettes had tried to quit smoking waterpipes some time in the past. - Nonsmokers held significantly stronger beliefs about the negative health effects of waterpipe than did smokers. - Waterpipe and cigarette smokers were the least likely to believe in the negative personal effects of waterpipe. - Those who smoked both waterpipe and cigarettes held the strongest beliefs about the positive personal benefits of smoking. - Waterpipe only smokers had significantly stronger beliefs about the positive personal benefits of waterpipe than nonsmokers. - Nonsmokers and cigarette smokers believed it was social pressures that encouraged people to smoke waterpipes, wheras the waterpipe only smokers and those who smoked both waterpipes and cigarettes did not believe social pressures was a cause of waterpipe use. |
| **Nakhostin-Roohi 2010**  **[**[**50**](#_ENREF_50)**]** | - Sampling frame: students from the Islamic Azad university- Ardabil branch (N: 10,433) - Sampling method: cluster sampling - Recruitment method: in person - Administration method: in person, self-administered | - Sample size calculation: no - Sampling type: probability sampling - Validity of tool: no validation reported - Pilot testing done: not reported - Response rate: 22.3% | - Country: Iran - Participants: university students from the Islami Azad university (mean age: 23.79, 55.9% males) - Waterpipe smokers: 35.6% - Setting: university, April to June 2009 - N sampled: 10,433 - N participated: 2,324 - N analyzed: 2,324 | **Motivations for smoking:**   - Relaxation (15.5%) – male 16.8%, female 11.9% - Recreation (59.7%) – male 55.8%, female 70.9% - Meeting (16.5%) – male 18.7%, female 10.3% - Other reasons (8.3%) – male 8.7%, female 6.9% |
| **Nakkash 2011 [**[**51**](#_ENREF_51)**]** | - [[51](#_ENREF_51)]Sampling frame: individuals living in urban areas of Beirut and rural areas of Bekaa (N: not reported) - Sampling method: purposive and snowball sampling - Recruitment method: in person - Administration method: in person, interviews and focus group discussions (FGD) | - Sample size calculation: no - Sampling method: non-probability sampling - Validity of tool: no validation reported - Pilot testing done: yes - Response rate: not reported | - Country: Lebanon - Participants: heavy waterpipe smokers in cafes, universities, and other public places in Beirut and Bekaa - Setting: various places in Lebanon, July and Sept to mid-Oct 2007 - N sampled: not reported - N participated: 220 - N analyzed: 220 | - Reasons for smoking: increased availability in the form of cafes, increased affordability, innovations in designs of waterpipe apparatus and tobacco flavours, sensory qualities (taste, smell, sight of smoke, sounds of bubbling), positive media portrayal of waterpipe - Commonly suggested policies: banning waterpipe indoors, raising prices, limit media advertising, need for health warnings. |
| **Poyrazoğlu 2010 [**[**52**](#_ENREF_52)**]** | - Sampling frame: students at Erciyes University (N:1866, 455 medical, 1411 engineering) - Sampling method: purposive sampling - Recruitment method: in person - Administration method: in person, self-administered with assistance when needed | - Sample size calculation: no - Sampling type: non-probability sampling - Validity of tool: self-developed, no validity reported - Pilot testing done: not reported - Response rate: 71.4% | - Country: Turkey - Participants: engineering and medical students from Erciyes University (mean age: 20.3, 57.8% males, 55% medical students) - Waterpipe smokers:   Current- 32.7%  Ever-45.1%   - Setting: university, 2008-2009 educational year - N sampled: 903 (455 medical, 448 engineering) - N participated: 651 - N analyzed: 645 | - Reasons listed for waterpipe smoking: enjoyment (72.4%), friend’s demand (12.4%), habit (0.5%), others (14.8%) - Respondents who are thinking of quitting: yes (21.0%), no (62.4%) - **Smokers vs. Non-Smokers:** - Perception of addictivity of waterpipe compared to cigarette smoking: similar (5.7% vs 9.4%, 8.2% total), less (65.2% v 31.0%, 42.2% total), more (14.8% vs 24.1%, 21.1% total), undecided (14.3% vs 35.4%, 28.5% total) - Perception of health damage of waterpipe compared to cigarette smoking to the smoker: similar (5.2% vs 8.7%, 7.6% total), less (23.3% vs 14.5%, 17.4% total), more (61.0% vs 49.7%, 53.5% total), undecided (10.5% vs 27.1%, 21.7%) - Perception of harmful effect of waterpipe compared to cigarette smoking to other people: similar (11.9% vs 11.3%, 11.5% total), less (28.6% vs 23.4%, 25.1% total), more (41.0% vs 27.6%, 31.9% total), undecided (18.6% vs 37.7%, 31.5% total) |
| **Radwan 2003 [**[**53**](#_ENREF_53)**]** | - Sampling frame: Household listing of a rural village (N = 305 households); listing of students in 2 secondary schools (N = not reported); patrons of 6 Shisha cafes in Cairo (N = not reported) - Sampling Method: systemic random sampling for the village; not reported for secondary schools and water pipe cafes. - Recruitment method: not reported - Administration method: household: In person, interviewer administered; school: In person, self-administered; shisha café: In person | - Sample size calculation: no - Sampling type: non-probability sampling - Validity of tool: self developed, validity not reported - Pilot testing: not reported - Response rate: not reported for rural adults and youths, 100% among water pipe café patrons, 73% among rural students | - Country: Egypt - Participants: rural village inhabitants, school’s students, water pipe cafes clients - Setting: houses, schools, and waterpipe cafes, timing not reported - N sampled: 2418 (households: 1483, secondary school students: 635, water pipe café clients: 300) - N participated: 2415 (households: 1161 adults and 322 youths, schools: 635, waterpipe cafes: 297) - N analyzed: 297 for shisha cafes, other settings: not reported | - A high level of belief that smoking is a sin: - rural adults: 97% - water pipe café patrons: 81% - rural youth 94% - rural students: 98% |
| **Roohafza 2011 [**[**54**](#_ENREF_54)**]** | - Sampling frame: students from Isfahan University and Kashan University (N: not reported) - Sampling method: simple random sampling - Recruitment method: not reported - Administration method: in person, interviewer administered | - Sample size calculation: no - Sampling type: probability sampling - Validity of tool: not reported - Pilot testing done: not reported - Response rate: 95% | - Country: Iran - Participants: university students, aged 20-25, who entered Isfahan University and Kashan University in 2007 (2.5% of girls and 18.3% of boys smoked cigarettes, 11.5% of girls and 28.7% of boys smoked ghelyan) - Setting: university, 2007 - N sampled: 855 - N participated: 812 - N analyzed: 233 (limited to smokers) | Perceived factors related to smoking initiation and maintenance-  **Females:**   - To decline anxiety: cig 3 (3, 27.3%), ghel 2 (3.8%) OR 10.25 (3.32-45.29) - Relief of stress: cig 5 (45.5%), ghel 6 (11.5%) OR 6.25 (2.14-18.17) - Decline a depressed mood: cig 1 (9.1%), ghel 1 (1.9%) OR 0.10 (0.37-8.12) - Facilitating greater concentration: cig 3 (27%), ghel 3 (5.7%), OR 7.59 (1.92-30.00) - Self-efficacy improvement: cig 1 (9.1%), ghel 3 (5.7%) OR 1.30 (0.01-13.15) - Achieving social acceptability: cig 1(9.1%), ghel 3 (5.7%), OR 2.52 (0.55-11.50) - Becoming mature: cig 1 (9.1%), ghel 3 (5.7%), OR 2.02 (0.34-11.84) - Being beloved: cig 1 (9.1%), ghel 4 (8.2%), OR 1.75 (0.41-7.44) - Hobby and fun: cig 5 (45.5%), ghel 42 (80.7%), OR 0.20 (0.07-0.56) - Pleasant sensation: cig 4 (36.4%), ghel 15 (28.8%), OR 1.45 (0.54-3.90) - Enjoying from tobacco odor: cig 3 (27.3%), ghel 31 (96.5%), OR 0.37 (0.22-0.90)   **Males:**   - To decline anxiety: cig 18 (27.2%), ghel 5 (4.8%) OR 7.24 (2.59-20.23) - Relief of stress: cig 24 (36.3%), ghel 23 (12.4%) OR 4.02 (1.92-8.42) - Decline a depressed mood: cig 15 (22.7%), ghel 4 (3.8%) OR 7.33 (2.27-22.66) - Facilitating greater concentration: cig 7 (10.6%), ghel 5 (4.8%), OR 2.53 (0.81-7.88) - Self-efficacy improvement: cig 9 (13.6%), ghel 2 (1.9%) OR 16.58 (2.09-51.34) - Achieving social acceptability: cig 27 (40.9%), ghel 20 (19.2%), OR 2.98 (1.54-5.77) - Becoming mature: cig 18 (27.3%), ghel 10 (9.6%), OR 3.83 (1.70-8.63) - Being beloved: cig 6 (9.1%), ghel 4 (5.7%), OR 1.50 (0.51-4.90) - Hobby and fun: cig 37 (56.1%), ghel 84 (80.7%), OR 0.32 (0.16-0.61) - Pleasant sensation: cig 21 (31.8%), ghel 31 (29.8%), OR 1.14 (0.61-2.15) - Enjoying tobacco odor: cig 20 (30.3%), ghel 60 (57.7%), OR 0.42 (0.25-0.95 |
| **Sabahy 2011 [**[**55**](#_ENREF_55)**]** | - Sampling frame: registrars of two major universities in Kerman (N: not reported) - Sampling method: simple random sampling - Recruitment method: not reported - Administration method: in person, self-administered | - Sample size calculation: no - Sampling type: probability sampling - Validity of tool: previously reported validated tool - Pilot testing done: not reported - Response rate: 91% | - Country: Iran - Participants: university students at two major universities in Kerman (mean age: 20.6, 50.5% female, 40% medical students) - Waterpipe smokers:   Current- 18.7%  Ever- 42.5%  Cigarette smokers: 11.8%   - Setting: university, timing not reported - N sample: 1130 - N participated: 1024 - N analyzed: 1024 | - The most important perceived stated reasons for waterpipe smoking: pleasure (55.8%), dealing with depression (22.1%), stress relief (9.0%), peer pressure (8.2%), and dealing with anger (4.9%) - The highest odds of ever smoking and current smoking of waterpipe were observed with having close friends who smoked the waterpipe (OR=5.94, p<0.001), being a current cigarette smoker (OR=2.40, p<0.002), and male sex (OR=1.87, p<0.001) |
| **Varsano 2003 [**[**56**](#_ENREF_56)**]** | - Sampling frame: school children in middle schools and high schools in Israel (N: not reported) - Sampling method: not reported - Recruitment method: not reported - Administration method: in person, self-administered with assistance of schoolteacher and nursing students | - Sample size calculation: no - Sampling type: not reported - Validity of tool:   Not reported   - Pilot testing: not reported - Response rate = not reported | - Country: Israel - Participants: children in middle school and high schools, aged 12-18 (males and females) - Waterpipe smokers: 41% (various frequencies) - Setting: schools, timing not reported - N sampled: 388 - N participated: 388 - N analyzed: 388 | - Main reasons for waterpipe smoking were pleasure achieved and the intimacy that it added to the youngster’s meetings - Belief that waterpipe smoking is not healthy: 90% - Belief that waterpipe smoking was less harmful than cigarettes: at least 50% |
| **Ward 2006 [**[**57**](#_ENREF_57)**]** | - Sampling frame: the municipality enumeration of Aleppo -Aleppo Household survey   (N: 2 million)   - Sampling Method: multistage sampling - Recruitment method: in person - Administration method : in person | - Sample size calculation: no - Sampling type: probability sampling - Validity of tool: self-developed tool, validation reported( from standardized questionnaires used in international settings) - Pilot testing: not reported - Response rate: 86% | - Country: Aleppo, Syria - Participants: residents of Aleppo, Syria (Mean age: 35.3, 45.2% males) - Waterpipe smoking   Males – 20.2%  Females – 4.8%   - Cigarette smoking   Males – 56.9%  Females – 17%   - Setting: community, 2004 - N sampled: 2370 - N participated: 2038 - N analyzed: 2038 | - Interest in quitting: waterpipe smokers (49%), cigarette smokers (74%) - Quit attempt in the past year: waterpipe smokers (23%), cigarette smokers (58%) (p < 0.0) - A central theme that emerged was that smoking water pipe is often viewed as an esthetic and sometimes even ecstatic experience that produces a sense of euphoria or close social inter-connectedness, while smoking cigarettes is viewed as a mundane addiction that provides some relief from anxiety in exchange for a persistent, nagging sense of being dominated |
| **Zoughaib 2004 [**[**58**](#_ENREF_58)**]** | - Sampling frame: 137 intermediate and secondary schools in Dahia, Lebanon   (N =75,000)   - Sampling Method: cluster random sampling - Recruitment method: in person - Administration: method: self- administered | - Sample size calculation: yes - Sampling type: probability sampling - Validity of tool: not reported - Pilot testing: yes - Response rate: not reported | - Country: Lebanon - Participants: teenagers in suburban schools (mean age: 15.7, 56.7% males) - Waterpipe smokers:   Ever – 66%   - Cigarette smokers:   Ever – 10.5%   - Setting: schools, school year 2001-2002 - N sampled: 1461 - N participated: 1461 - N analyzed: 1461 | - Belief that their parents either somewhat or strongly opposed the use or narghile: 62.8% - No desire to quit the habit: 44% - Perception that narghile is less harmful than cigarettes: 43%% - Belief that narghile use was harmless to health: 1% - Perception that waterpipe is less harmful than cigarette smoking significantly higher in regular users compared to all other categories of use (occasional, initiators, non-starters) (P< 0.01). |

**References**

1. Abughosh S: **Predictors of Persisten Waterpipe Smoking Among University Students in The United Students**. *Epidemiology* 2011, **1**(1).

2. Ahmed B, Jacob P, 3rd, Allen F, Benowitz N: **Attitudes and practices of hookah smokers in the San Francisco Bay Area**. *J Psychoactive Drugs* 2011, **43**(2):146-152.

3. Aljarrah K, Ababneh ZQ, Al-Delaimy WK: **Perceptions of hookah smoking harmfulness: Predictors and characteristics among current hookah users**. *Tobacco Induced Diseases* 2009, **5**(1).

4. Braun R: **Hookah Use Among College Students from a Midwest University**. *Journal of Community Health* 2011:1-5.

5. Cheron-Launay M, Baha M, Mautrait C, Lagrue G, Le Faou AL: **[Identifying addictive behaviors among adolescents: a school-based survey]**. *Arch Pediatr* 2011, **18**(7):737-744.

6. Combrink A, Irwin N, Laudin G, Naidoo K, Plagerson S, Mathee A: **High prevalence of hookah smoking among secondary school students in a disadvantaged community in Johannesburg**. *Samj, S* 2010, **100**(5):297-299.

7. Dillon KA, Chase RA: **Secondhand smoke exposure, awareness, and prevention among African-born women**. *Am J Prev Med* 2010, **39**(6 Suppl 1):S37-43.

8. Eissenberg T, Ward KD, Smith-Simone S, Maziak W: **Waterpipe tobacco smoking on a US college campus: Prevalence and correlates**. *Journal of Adolescent Health* 2008, **42**(5):526-529.

9. Giuliani KKW, Mire O, Ehrlich LC, Stigler MH, Dubois DK: **Characteristics and prevalence of tobacco use among Somali youth in Minnesota**. *Am J Prev Med* 2010, **39**(6 Suppl 1):S48-55.

10. Griffiths MA, Harmon TR, Gilly MC: **Hubble Bubble Trouble: The Need for Education About and Regulation of Hookah Smoking**. *J Public Policy Mark* 2011, **30**(1):119-132.

11. Jackson D, Aveyard P: **Waterpipe smoking in students: Prevalence, risk factors, symptoms of addiction, and smoke intake: evidence from one British university**. *BMC Public Health* 2008, **8**:5.

12. Jamil H, Elsouhag D, Hiller S, Arnetz JE, Arnetz BB: **Sociodemographic risk indicators of hookah smoking among White Americans: a pilot study**. *Nicotine & Tobacco Research* 2010, **12**(5):525-529.

13. Jamil H, Janisse J, Elsouhag D, Fakhouri M, Arnetz JE, Arnetz BB: **Do household smoking behaviors constitute a risk factor for hookah use?** *Nicotine & Tobacco Research* 2011, **13**(5):384-388.

14. Lipkus IM, Eissenberg T, Schwartz-Bloom RD, Prokhorov AV, Levy J: **Affecting perceptions of harm and addiction among college waterpipe tobacco smokers**. *Nicotine & Tobacco Research* 2011, **13**(7):599-610.

15. Perusco A, Rikard-Bell G, Mohsin M, Millen E, Sabry M, Poder N: **Tobacco control priorities for Arabic speakers: key findings from a baseline telephone survey of Arabic speakers residing in Sydney's south-west.** *Health Promot J Austr* 2007, **18**(2):121-126.

16. Carroll T, Poder N, Perusco A: **Is concern about waterpipe tobacco smoking warranted ?** *Australian and New Zealand Journal of Public Health* 2008, **32**(2):181-U111.

17. Primack B, Sidani J, Agarwal A, Shadel W, Donny E, Eissenberg T: **Prevalence of and Associations with Waterpipe Tobacco Smoking among U.S. University Students**. *Annals of Behavioral Medicine* 2008, **36**(1):81-86.

18. Richter PA, Pederson LL, O'Hegarty MM: **Young adult smoker risk perceptions of traditional cigarettes and nontraditional tobacco products**. *Am J Health Behav* 2006, **30**(3):302-312.

19. Roskin J, Aveyard P: **Canadian and English students' beliefs about waterpipe smoking: a qualitative study**. *BMC Public Health* 2009, **9**(1):10.

20. Smith SY, Curbow B, Stillman FA: **Harm perception of nicotine products in college freshmen**. *Nicotine & Tobacco Research* 2007, **9**(9):977 - 982.

21. Smith JR, Novotny TE, Edland SD, Hofstetter CR, Lindsay SP, Al-Delaimy WK: **Determinants of hookah use among high school students**. *Nicotine & Tobacco Research* 2011, **13**(7):565-572.

22. Smith-Simone S, Maziak W, Ward KD, Eissenberg T: **Waterpipe tobacco smoking: Knowledge, attitudes, beliefs, and behavior in two US samples**. *Nicotine & Tobacco Research* 2008, **10**(2):393-398.

23. Smith-Simone SY, Curbow BA, Stillman FA: **Differing psychosocial risk profiles of college freshmen waterpipe, cigar, and cigarette smokers**. *Addict Behav* 2008, **33**(12):1619-1624.

24. Sutfin EL, McCoy TP, Reboussin BA, Wagoner KG, Spangler J, Wolfson M: **Prevalence and correlates of waterpipe tobacco smoking by college students in North Carolina**. *Drug Alcohol Depend* 2011, **115**(1-2):131-136.

25. Ward KD, Eissenberg T, Gray JN, Srinivas V, Wilson N, Maziak W: **Characteristics of US waterpipe users: A preliminary report**. *Nicotine & Tobacco Research* 2007, **9**(12):1339-1346.

26. Weglicki LS, Templin T, Hammad A, Jamil H, Abou-Mediene S, Farroukh M, Rice VH: **Tobacco use patterns among high school students: Do Arab American youth differ?** *Ethnicity & disease* 2007, **17**((2 Suppl 3)):S3-22-S23-24.

27. Afifi RA, Yeretzian JS, Rouhana A, Nehlawi MT, Mack A: **Neighbourhood influences on narghile smoking among youth in Beirut**. *Eur J Public Health* 2010, **20**(4):456-462.

28. Al-Dabbagh S, Al-Sinjari KM: **Knowledge, attitude and believes of Nargila (hubble-bubble) smoking in Iraq.** . *Journal of the Bahrain Medical Society* 2005.

29. Allam MF, Abd Elaziz KM: **Role of members of university students' unions in tobacco prevention**. *J* 2007, **48**(4):136-140.

30. Almerie MQ, Matar HE, Salam M, Morad A, Abdulaal M, Koudsi A, Maziak W: **Cigarettes and waterpipe smoking among medical students in Syria: a cross-sectional study**. *Int J Tuberc Lung Dis* 2008, **12**(9):1085-1091.

31. Anjum Q, Ahmed F, Ashfaq T: **Knowledge, attitude and perception of water pipe smoking (Shisha) among adolescents aged 14-19 years**. *JPMA J Pak Med Assoc* 2008, **58**(6):312-317.

32. Aydin N, Uyar M, Kul S, Elbek O: **Awareness of teachers towards the law "4207"**

**Ogretmenlerin 4207 sayi{dotless}li{dotless} yasa konusundaki farki{dotless}ndali{dotless}klari{dotless}**. *TAF Preventive Medicine Bulletin* 2011, **10**(5):543-548.

33. Azab M, Khabour OF, Alkaraki AK, Eissenberg T, Alzoubi KH, Primack BA: **Water pipe tobacco smoking among university students in Jordan**. *Nicotine & Tobacco Research* 2010, **12**(6):606-612.

34. Chaaya M, Jabbour S, El-Roueiheb Z, Chemaitelly H: **Knowledge, attitudes, and practices of argileh (water pipe or hubble-bubble) and cigarette smoking among pregnant women in Lebanon**. *Addict Behav* 2004, **29**(9):1821-1831.

35. Dar-Odeh NS, Bakri FG, Al-Omiri MK, Al-Mashni HM, Eimar HA, Khraisat AS, Abu-Hammad SMK, Dudeen AAF, Abdallah MN, Alkilani SMZ *et al*: **Narghile (water pipe) smoking among university students in Jordan: prevalence, pattern and beliefs**. *Harm Reduct J* 2010, **7**.

36. Erbaydar NP, Bilir N, Yildiz AN: **KNOWLEDGE, BEHAVIORS AND HEALTH HAZARD PERCEPTION AMONG TURKISH NARGHILE (WATERPIPE)-SMOKERS RELATED TO NARGHILE SMOKING**. *Pak J Med Sci* 2010, **26**(1):195-200.

37. Ghafouri N, Hirsch J, Heydari G, Morello C, Kuo G, Singh R: **Waterpipe smoking among health sciences university students in Iran: perceptions, practices and patterns of use**. *BMC Research Notes* 2011, **4**(1):496.

38. Hammal F, Mock J, Ward K, Eissenberg T, Maziak W: **A pleasure among friends: how narghile (waterpipe) smoking differs from cigarette smoking in Syria**. *Tob Control* 2008, **17**(2):e3.

39. Israel E, El-Setouhy M, Gadalla S, Aoun el SA, Mikhail N, Mohamed MK, Israel E, El-Setouhy M, Gadalla S, Aoun ESA *et al*: **Water pipe (Sisha) smoking in cafes in Egypt**. *Journal of the Egyptian Society of Parasitology* 2003, **33**(3 Suppl):1073-1085.

40. Jawaid A, Zafar A, Rehman T, Nazir M, Ghafoor Z, Afzal O, al. e: **Knowledge, attitudes, and practice of university students regarding waterpipe smoking in Pakistan**. *Int J Tuberc Lung Dis* 2008, **12**(9):1077-1084.

41. Kelishadi R, Mokhtari MR, Tavasoli AA, Khosravi A, Ahangar-Nazari I, Sabet B, Kazemi A, Amini A, Kelishadi R, Mokhtari MR *et al*: **Determinants of tobacco use among youths in Isfahan, Iran**. *Int J Public Health* 2007, **52**(3):173-179.

42. Labib N, Radwan G, Mikhail N, Mohamed MK, El Setouhy M, Loffredo C, Israel E: **Comparison of cigarette and water pipe smoking among female university students in Egypt**. *Nicotine & Tobacco Research* 2007, **9**(5):591-596.

43. Makhoul J, Nakkash R: **Understanding youth: using qualitative methods to verify quantitative community indicators**. *Health Promotion Practice* 2009, **10**(1):128-135.

44. Maziak W, Rastam S, Eissenberg T, Asfar T, Hammal F, Bachir ME, Fouad MF, Ward KD: **Gender and smoking status-based analysis of views regarding waterpipe and cigarette smoking in Aleppo, Syria**. *Preventive Medicine* 2004, **38**(4):479-484.

45. Maziak W, Eissenberg T, Rastam S, Hammal F, Asfar T, Bachir ME, Fouad MF, Ward KD: **Beliefs and attitudes related to narghile (waterpipe) smoking among university students in Syria**. *Annals of Epidemiology* 2004, **14**(9):646-654.

46. Maziak W, Ward KD, Eissenberg T, Maziak W, Ward KD, Eissenberg T: **Factors related to frequency of narghile (waterpipe) use: the first insights on tobacco dependence in narghile users**. *Drug & Alcohol Dependence* 2004, **76**(1):101-106.

47. Ward KD, Hammal F, VanderWeg MW, Eissenberg T, Asfar T, Rastam S, Maziak W: **Are waterpipe users interested in quitting?** *Nicotine & Tobacco Research* 2005, **7**(1):149-156.

48. Asfar T, Ward KD, Eissenberg T, Maziak W, Asfar T, Ward KD, Eissenberg T, Maziak W: **Comparison of patterns of use, beliefs, and attitudes related to waterpipe between beginning and established smokers**. *BMC Public Health* 2005, **5**:19.

49. Mohammed HR, Zhang Y, Newman IM, Shell DF: **Waterpipe smoking in Kuwait**. *East Mediterr Health J* 2010, **16**(11):1115-1120.

50. Nakhostin-Roohi B, Valizadeh S: **Hookah smoking in students: Prevalence, pattern of smoking, situational characteristics and motivation of use: Evidence from one Iranian university**. *Gazzetta Medica Italiana Archivio per le Scienze Mediche* 2010, **169**(2):41-45.

51. Nakkash RT, Khalil J, Afifi RA: **The rise in narghile (shisha, hookah) waterpipe tobacco smoking: a qualitative study of perceptions of smokers and non smokers**. *BMC Public Health* 2011, **11**:315.

52. Poyrazoglu S, Sarli S, Gencer Z, Gunay O: **Waterpipe (narghile) smoking among medical and non-medical university students in Turkey**. *Ups J Med Sci* 2010, **115**(3):210-216.

53. Radwan GN, Israel E, El-Setouhy M, Abdel-Aziz F, Mikhail N, Mohamed MK, Radwan GN, Israel E, El-Setouhy M, Abdel-Aziz F *et al*: **Impact of religious rulings (Fatwa) on smoking**. *Journal of the Egyptian Society of Parasitology* 2003, **33**(3 Suppl):1087-1101.

54. Roohafza H, Sadeghi M, Shahnam M, Bahonar A, Sarafzadegan N: **Perceived factors related to cigarette and waterpipe (ghelyan) initiation and maintenance in university students of Iran**. *Int J Public Health* 2011, **56**(2):175-180.

55. Sabahy AR, Divsalar K, Bahreinifar S, Marzban M, Nakhaee N: **Waterpipe tobacco use among Iranian university students: correlates and perceived reasons for use**. *Int J Tuberc Lung Dis* 2011, **15**(6):844-847.

56. Varsano S, Ganz I, Eldor N, Garenkin M, Varsano S, Ganz I, Eldor N, Garenkin M: **[Water-pipe tobacco smoking among school children in Israel: frequencies, habits, and attitudes]**. *Harefuah* 2003 **142**(11):736-741.

57. Ward KD, Eissenberg T, Rastam S, Asfar T, Mzayek F, Fouad MF, Hammal F, Mock J, Maziak W: **The tobacco epidemic in Syria**. *Tob Control* 2006, **15**:I24-I29.

58. Zoughaib SS, Adib SM, Jabbour J, Zoughaib SS, Adib SM, Jabbour J: **Prevalence and determinants of water pipe or narghile use among students in Beirut's southern suburbs**. *Journal Medical Libanais - Lebanese Medical Journal* 2004, **52**(3):142-148.
